# Exploring the Application of Micellar Drug Delivery Systems in Cancer Nanomedicine

**DOI:** 10.3390/ph16030433

**Published:** 2023-03-12

**Authors:** Qi Wang, Keerthi Atluri, Amit K. Tiwari, R. Jayachandra Babu

**Affiliations:** 1 Department of Drug Discovery and Development, Auburn University, Auburn, AL 36849, USA; 2 Product Development Department, Alcami Corporation, Morrisville, NC 27560, USA; 3 Department of Pharmacology and Experimental Therapeutics, University of Toledo, Toledo, OH 43614, USA; 4 Department of Cell and Cancer Biology, University of Toledo, Toledo, OH 43614, USA

**Keywords:** micelle, polymer, drug delivery, stimuli sensitive, regulatory, clinical trials

## Abstract

Various formulations of polymeric micelles, tiny spherical structures made of polymeric materials, are currently being investigated in preclinical and clinical settings for their potential as nanomedicines. They target specific tissues and prolong circulation in the body, making them promising cancer treatment options. This review focuses on the different types of polymeric materials available to synthesize micelles, as well as the different ways that micelles can be tailored to be responsive to different stimuli. The selection of stimuli-sensitive polymers used in micelle preparation is based on the specific conditions found in the tumor microenvironment. Additionally, clinical trends in using micelles to treat cancer are presented, including what happens to micelles after they are administered. Finally, various cancer drug delivery applications involving micelles are discussed along with their regulatory aspects and future outlooks. As part of this discussion, we will examine current research and development in this field. The challenges and barriers they may have to overcome before they can be widely adopted in clinics will also be discussed.

## 1. Introduction

Cancer continues to be one of the leading causes of death throughout the world, despite extensive research and advances in treatment. With nanotechnology, materials can be manipulated and engineered at the nanometer scale, revolutionizing cancer treatment. To better understand how nanotechnology can be applied to the diagnosis and how to deliver chemotherapy drugs directly to cancer cells for targeted drug delivery systems is currently the subject of intense study. Drug transport, imaging, immune system development, diagnostics, and therapy all benefit from the use of nanomaterials. Several nanomaterials, such as liposomes and polymeric micelles used for the treatment of cancer, have been approved by regulatory authorities in several countries, including the United States and Europe, and a few other nanomedicines are currently under clinical investigation. Hypoxia, acidosis, and vascular anomalies are some of the features that the tumor microenvironment (TME) differs from normal tissues [1,2].

Low pH, high glutathione (GSH) concentrations, excess production of reactive oxygen species (ROS), and severe hypoxia are some of the typical features of TME. Tumor development, metastasis, and medication resistance may result from tumors with these features because they create a conducive internal environment for tumor cells to survive. These features could be used to develop targeted nanomedicine delivery systems that selectively release drugs only in tumor tissues with minimal systemic drug exposures. Stimuli responsive nanoparticles can release drugs only in response to certain stimuli, prolonging blood circulation and enhancing cancer cell absorption while also improving biosafety. They can also maintain stability under physiological conditions. As a result, there is great potential that the development of TME-responsive smart nanomedicine may improve the efficiency of existing cancer treatments [3,4].

### 1.1. Targeting TME with a Low pH 

Although the extracellular pH of healthy tissue is carefully controlled at around 7.4, it is frequently dysregulated in pathological conditions such as ischemia, inflammation, and neoplasia. Due to tumor cell anaerobic glycolysis and lactic acid generation, the TME is generally acidic. Tumors prefer anaerobic glycolysis even when exposed to oxygen, a phenomenon known as the Warburg effect [5]. Numerous investigations have demonstrated that the unregulated energy metabolism, inadequate perfusion, and accumulation of lactic acid (Warburg effect) cause the extracellular space of tumor tissue to be weakly acidic, with a pH range from 6.5 to 6.8 [6,7,8]. Recent studies have shown the development of pH responsive nanomedicines due to the high acidity in the tumor extracellular environment being a characteristic pathological hallmark of solid tumor tissues in comparison to the neutral environment of normal tissues. Therefore, acidity permeates the TME, and delivery devices targeting low extracellular pH would permit very selective delivery of cargo to the tumors in vivo [9,10,11].

### 1.2. Targeting TME with High Level of GSH 

GSH is a thiol compound made of cysteine, glutamate, and glycine. It is essential for the body to have a normal concentration of GSH because it has antioxidant and detoxifying properties [6]. To keep the cellular redox state in check, GSH is an essential component as one of the most prevalent reductive cellular metabolites. GSH mediates the formation and breakdown of disulfide bonds, making it an important player in the regulation of protein folding. The reported GSH content in tumor cells is significantly greater than that in normal cells. About 2–10 mM of GSH is present intracellularly, which is a considerable increase over the 2–20 M levels found in the extracellular matrix and blood. Additionally, compared to normal tissues, tumor tissues had a GSH concentration that was ten times higher [3,12,13]. As oxidized glutathione (GSSG) is catabolized in the cytosol into GSH by GSH reductases and nicotinamide adenine dinucleotide phosphate (NADP), the cytosol contains 1000 times more GSH than the surrounding environment or plasma. For this reason, disulfide bonds have been included in nanomedicine to promote selective drug release in the tumor cytosol via GSH as a particular marker [6,14,15].

### 1.3. Targeting Hypoxia TME

Hypoxia is a defining feature of solid tumors and is intimately associated with invasion, metastasis, and medication resistance. Blood arteries in solid tumors are unable to adequately distribute oxygen and nutrients to all areas due to their uneven structure, which causes tumor cells to become hypoxic temporarily or permanently. From the tumor surface to the center, the oxygen partial pressure gradually drops. The oxygen partial pressure in some locations can be as low as 0–2.5 mmHg, which creates a hypoxic environment around the tumor compared to the normal tissue’s 30–40 mmHg oxygen partial pressure [3,6,16]. The oxygen partial pressure in normal tissues is around 30 mmHg, whereas it steadily drops from the outside to the inside of tumor tissues and reaches a low level (5 mmHg) in some areas; and in some solid tumors, it may even be close to 0 mmHg. Hypoxia in the TME can upregulate hypoxia-inducible factors (HIFs), a protein dimerization made up of the HIF-(oxygen-sensitive subunit) and HIF-(constitutively expressed subunit) subunits that can promote tumor growth and metastasis. This hypoxic adaptation influences activities like cell energy metabolism, endocytic receptor internalization, transmembrane receptor recirculation, and transportation by altering the general biochemical environment around cells [3,17,18]. Hypoxia is becoming the main focus of both diagnosis and treatment because of the obvious differences between tumor tissue and normal tissue. In order to treat and image tumors, hypoxia can therefore be exploited as an endogenous stimulation. Quinone, nitroaromatic, and azobenzene derivatives are the principal functional groups that react to hypoxia and have been widely used as hypoxia-responsive nanomedicine or nanoprobes [6,19,20,21,22]. Hypoxia sensitive polymers have shown potential in developing trigger-release nanomedicine responses to specific TME conditions.

### 1.4. Targeting TME with High Level of ROS

The concentration of ROS, which is 2–5 times greater in tumor tissues than in normal tissues, has been reported. The production of ROS, particularly hydrogen peroxide (H_2_O_2_), is essential for several physiological activities [23,24]. Through processes involving the mitochondrial respiratory chain and nicotinamide adenine dinucleotide phosphate oxidase, the majority of tumor cells create more ROS than normal cells. Based on the high amounts of ROS in tumor tissues, many ROS-responsive polymers have been investigated. These include unsaturated lipids, sulfur, selenium, tellurium, and other ROS-sensitive groups. High ROS level has been used as a stimulus to trigger drug release from nanomedicine in TME [25,26]. 

### 1.5. Targeting Specific Enzymes in TME

Some enzymes are overexpressed in tumor tissues compared to regular tissues. Matrix metalloproteinases (MMPs), hyaluronidase, glucosidase, and esterase are all examples of enzymes that are oversecreted in the TME. Drugs can be released in the TME from nanomedicines by having them modified with enzyme substrates [3,27,28]. MMPs, which are overexpressed in the extracellular environment of many malignancies, are an attractive target for drug release triggers [29].

### 1.6. The Enhanced Permeability and Retention (EPR) Effect and Its Application in Nanomedicine Delivery 

To be effective, nanomedicines need to do more than simply circulate throughout the body. Thus, the preferential accumulation of nanomedicines in solid tumors is important for the advancement of anticancer therapy. The EPR effect increases the retention of the macromolecules and mediates the prominent accumulation of drug carriers in tumors. With the systemic injection, long-circulating PEGylated nanoparticles have a better chance of targeting tumors due to the increased EPR effect. Circulating nanomedicines can preferentially concentrate in tumors because the apertures prevalent throughout the tumor vasculature (>400 nm) are considerably greater than those of endothelial fenestrae in the liver [30,31,32]. Tumors have a varied blood supply and permeability, which may induce an inhomogeneity in the delivery of nanomedicines across the tumor sites. The EPR effect is the primary mechanism through which nanoparticles accumulate in tumor tissue. Nanoparticles have more difficulty moving through the vasculature of any given tissue, whereas tiny molecules can do so with relative ease. Because tumors and healthy tissues have distinct vascular networks, malignancies are a major source of the EPR effect [33]. Therefore, the carriers should have a prolonged blood half-life and successful extravasation and deep penetration from the blood compartment into tumor tissues, as well as have the ability to exploit the EPR effect for tumor targeting and uniformly distribute adequate dosages of drugs [34,35]. 

### 1.7. Micelles as Nanomedicine Delivery Systems

The nanomedicine approach to delivering hydrophobic drugs is becoming a common and effective strategy for overcoming the challenges associated with drug delivery. In order to achieve the desired therapeutic response, an adequate quantity of the active drug must arrive at the site of action, and the effective concentration of the drug must be kept at the target location for a predetermined amount of time. However, this process is hampered for the vast majority of drugs due to several obstacles. These include rapid degradation of drugs in an in vivo environment, inadequate pharmacokinetics (PK), a lack of selectivity for the tissues that are being targeted, and the possibility of systemic toxicity [34]. In order to encapsulate drugs, nanoparticulate medicines such as polymer-based micelles have been utilized. Nanomedicines have the potential to accomplish both sustained circulation and accumulation at the target site [36]. 

To form polymeric micelles, biocompatible synthetic polymers or natural macromolecules can be used. The core-forming segments are primarily responsible for determining important aspects of the polymeric micelle, such as shape, stability, drug-loading capacity, and drug-release profile [37,38]. When compared to conventional drug delivery systems, the nanosize of the micelles makes it possible for the drug to extravasate via the leaky vasculature more effectively. The hydrophilic polymeric coating will make it possible for them to avoid being detected by the reticuloendothelial system while they are in circulation. Because of the hydrophilic shell of the micelle, nanoparticles are able to maintain their steric stability and experience less non-specific absorption by the reticuloendothelial system (RES). This results in a longer period of time spent circulating throughout the body. In order for micelles to be successfully delivered, they need to maintain a steady circulation in the blood compartment while avoiding undesired interactions with blood components and the RES. Micelles should also selectively extravasate at the sick (tumor) location, where the target cells can pick them up and release them intracellularly. Micelles should also extravasate at the diseased site. Micelles can be administered directly into the bloodstream, which allows for rapid and uniform distribution throughout the body. This route is often used for cancer treatments, as it allows for targeted delivery of chemotherapy drugs to tumor sites. The other routes of administration such as oral, transmucosal or topical administration are utilized to deliver micellar formulation for various localized non-cancer diseases.

Nanomedicine with a macromolecular or particulate nature can aggregate in the tumor tissues and remain in the TME in order to prolong its retention time at the target location since the arterial walls are damaged and leaky, and the lymphatic drainage is poor. Over the course of the last few decades, various types of micelles have been explored for their potential use in the administration of chemotherapy drugs in cancer therapy [39,40,41,42]. Though various types of stimuli responsive micelles have been reported in the literature for various therapeutic uses, the materials that are used in the construction of micelles in recent years are not well documented, which is the focus of this review. Furthermore, this review provides recent updates on the clinical trials and procedures related to regulatory submissions for micellar nanocarrier systems.

## 2. Characteristic Features of Micelles

Because of the mechanical and physical properties that they possess, certain polymers and surfactants have the ability to self-assemble into specific systems. The fact that it puts itself together can help the structure be more stable. The concentration at which the polymers or surfactants prefer to assemble themselves in ordered micellar structures is referred to as the Critical Micelle Concentration (CMC). Since the surface tension of a solution is affected by the concentration of the polymer in the solution, it is possible to utilize surface tension to calculate CMC. The fluorometric method, the approach based on surface tension, the method based on light scattering, the method based on electric conductivity, the method based on osmotic pressure, the method based on the surface plasmon resonance, and the method based on electric conductivity are some of the more common methods used to determine CMCs. Micelles are formed through the self-assembly of amphiphilic polymers at the CMC. The Krafft point, also known as critical micelle temperature (CMT), is the minimum temperature at which the detergent will form micelles. At its CMT, the solubility of a surfactant is equal to its critical micelle concentration, indicating that the surfactant can form micelles. For detergents, insolubility causes precipitation at temperatures below CMT at or above the detergent’s CMC. Micelles are produced when block copolymers, random block copolymers, and grafted polymers self-assemble into their desired structures. Transmission electron microscopy, atomic force microscopy, small-angle neutron scattering, small-angle X-ray scattering, dynamic light scattering, and electron paramagnetic resonance spectroscopy are some of the methods that are used to characterize the micelles [43,44,45,46,47,48].

**Figure 1:**
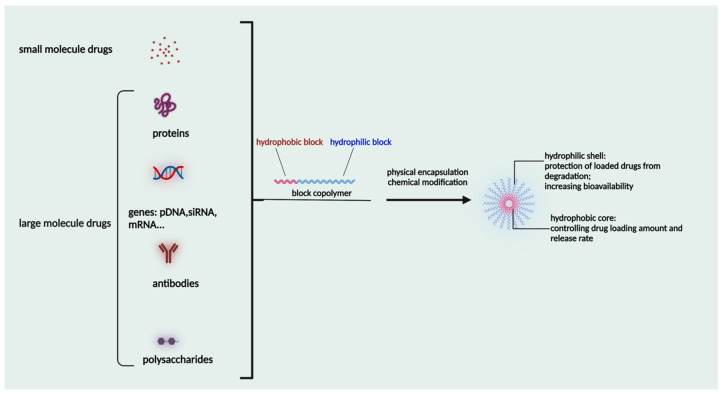
Structure of polymeric micelles for loading small and large molecule drugs. Created with BioRender.com.

Polymeric micelles are self-assembled in aqueous environments from amphiphilic polymers, which are the building blocks of polymeric micelles, as shown in Figure 1. The construction of these amphiphilic polymers involves the use of a variety of polymeric building components. The blocks are able to be customized depending on the need for an optimal balance of hydrophobic and lipophilic groups, size, drug loading capability, micellization ability, and stability in the systemic circulation. When the concentration reaches CMC or even higher, the amphiphilic polymers will self-assemble into micelles in the shape of spheres. The brush-like structure of the head, which is hydrophilic, combines to form the shell, while the hydrophobic tail aggregates to form the inner core of the structure. Through the use of hydrophobic interactions, hydrophobic drugs can be contained inside this core. The hydrophilic units that are present in the micelle’s shell will engage in interactions with the water molecules that are in its immediate environment. The micelles are highly stable in the liquid state in the aqueous solution [43,49,50]. The size and shape of micelles can be analyzed by scanning electron microscopy and transmission electron microscopy. 

The drug loading is affected by its hydrophobic interaction with the micellar core, as well as polar interactions and hydrogen bonding to some extent [51]. The drug loading efficiency can also be affected by the hydrophobic block chain length, the substituted groups, and the block copolymer aggregates [52]. The micellar structures in the physiological environment should be stable and long circulating to enable their uptake by the tumor tissue and should not cause any side effects during their fate in the body. 

The physical stability of micelles is dependent on the CMC, which is determined by the hydrophilic and hydrophobic nature of the polymer, and polymers with long hydrophobic chains show lower stability. The physical state of the micelle core, amount of solvent, size of the hydrophobic block, and hydrophobic/hydrophilic balance of the polymer determine the physical stability of the micelles. The physical stability of polymeric micelles can be higher for materials with low CMC values. Increased intra-micellar interactions and covalent cross-linking of the micelle core can also increase physical stability [53]. The drug loading efficiency and physical stability were increased by attaching fatty acids to the core of polyethylene oxide-poly(aspartic acid) (PEO-P(Asp)) micelles, modifying the core with structures capable of forming intra-micellar structures and electrostatic interactions, and covalent cross-linking [52,54].

## 3. Polymers Used for Micelle Formation

The assembly of the block copolymer, the arrangement of the polymers, the stability of micelles, and their biodistribution are all determined by the segments of the block copolymer. When choosing polymers, on the other hand, it is important to take into account the structure of the micelle complex as well as the inherent safety of these polymers. These polymers make it possible for micelles to dissolve and be expelled from the body, hence preventing any adverse long-term effects [41]. The covalent binding of drugs to polymers that dissolve in water can extend their half-life in the bloodstream and reduce their toxicity to healthy cells and tissue. Polymers have been modified by including polyethylene glycol (PEG) to prevent opsonization and lengthen their circulation time, incorporating targeting ligands, and employing pH-sensitive or hypothermic polymer conjugates [55,56]. 

PEG is the most popular and most effective stealth polymer in the field of polymer-based drug delivery is PEG. Twenty years have passed since the introduction of the first PEGylated products for sale. Hypersensitivity, unexpected changes in pharmacokinetic behavior, toxic side products, an antagonism arising from the easy degradation of the polymer, and the resulting possible accumulation in the body may all need consideration. PEG is very soluble in organic solvents, making it simple to modify its end groups. PEG is an excellent polymer for use in biological systems, since it is soluble in water and has a low intrinsic toxicity. The hydrophilicity of PEG improves the solubility of hydrophobic medicines or carriers in water. It improves the physical and thermal stability of pharmaceuticals and eliminates or greatly decreases drug aggregation in vivo and in storage [57,58]. Polysaccharides are a wide variety of polymeric substances with natural origins, known as polysaccharides. Polysaccharides are found naturally, are renewable, pose no health risks, and break down quickly. They are created through the glycosidic bonding of monosaccharides. The architecture of polysaccharides can be either linear or branching, depending on the type of monosaccharide unit. Polysaccharides have a variety of reactive groups, such as hydroxyl, amino, and carboxylic acid groups, which further suggests the potential for chemical alteration. These functional groups can be used to modify polysaccharides with small molecules, polymers, and crosslinkers, and the resulting modified polysaccharides have proven to be useful building blocks in the development of novel biomaterials for use in a wide range of biomedical settings, including as drug delivery carriers, cell-encapsulating biomaterials, and tissue engineering scaffolds. Further increasing diversity, polysaccharide molecular weight can range from hundreds to thousands of Daltons. The majority of polysaccharides are susceptible to enzymatic breakdown as a result of their natural existence in the body. Polysaccharides can be recycled for use as storage, structural support, or even cell signaling by breaking them down into their monomer or oligomer building parts through enzyme catalysis [59,60,61,62,63,64]. Regarding Poly[N-(2-hydroxypropyl) methacrylamide], pHPMA, research into pHPMA’s potential as a polymeric micelle building block has focused on both its use as a hydrophilic shell component and its use as a hydrophobic core derivative. As a potential building block of polymeric micelles with a hydrophilic, shell-forming property, pHPMA is a promising contender. In comparison to PEG, pHPMA is advantageous due to its multifunctionality, which permits the conjugation of numerous therapeutic or targeting molecules to a single polymer chain without compromising biocompatibility or non-immunogenicity [65,66,67,68]. Regarding Poly(amino acids), because of their many useful properties, including biodegradability, biocompatibility, and the availability of side functional groups, poly(amino acid) and its derivatives are commonly used to form polymeric micelles. These materials have several applications due to their many desirable properties, including biodegradability, biocompatibility, and a high number of side functional groups. With the right design of hydrophobic segments and the right amount of side functional groups, these polymeric micelles often exhibit a high drug loading capacity for both hydrophobic and hydrophilic agents. Due to the adaptability of their chemical structures and the availability of functional groups on polymer, micelles of amphiphilic poly(amino acid) copolymers can load a wide variety of potential pharmaceuticals through non-covalent contact. Engineering of the polymeric structure also allows for a high drug loading capacity in these micelles. Special strategies, such as crosslinking and layer-by-layer coating, may thereby further stabilize the drug-loaded micelles in terms of physical loading [69,70,71]. Regarding polyethers, developments in polyether-based amphiphilic nanocarriers have made it possible to easily distribute active components while avoiding the toxicity, unwanted side effects, and hypersensitivity reactions associated with conventional surfactants. Delivering active components at high dilutions in the bloodstream is now possible thanks to the low CMCs of these nanocarriers. PEG often conjugates with these polyethers. The PEG-based amphiphilic nanocarriers show optimal biocompatibility over cellular and systemic levels. They may have drawbacks, including degradation under stress, accumulation in the body above an uncertain excretion limit, and interaction with the immune system. Since PEG has very uninteresting end group functionality, there is not much room for alteration at the polyether backbone to modify [34,72]. Regarding polyesters (such as poly(L, D-lactide), PLA), PLA has the mechanical and physical qualities that may be designed to fit a wide variety of uses, and PLA is also biodegradable via hydrolysis and enzymatic activity, and it has a low immunogenicity characteristic. The Food and Drug Administration (FDA) has authorized various formulations incorporating PLA, further demonstrating its suitability for rapid clinical translation. These biomaterials can be made into a wide variety of useful products, including sutures, scaffolds, cell carriers, medication delivery systems, and more. Numerous studies, both laboratory and human, have been conducted on PLA. Liposomes, polymeric nanoparticles, dendrimers, and micelles are only a few of the nanoparticle drug carriers that can be loaded with PLA to encapsulate hydrophobic anti-tumor medicines and protect the body from their systemic toxicity. It is an ever-evolving discipline that sees modest improvements in the clinical translation of these technologies from preclinical experimental settings [73,74]. A summary of the polymers commonly used for polymeric micelles is listed in Table 1 and Table 2. 

## 4. Micelles in Tumor Targeted Drug Release

Polymeric micelles are a novel type of drug delivery system that offers a variety of advantages. These advantages include fewer adverse effects of systematic toxicity, more selective targeting to specific tissues due to stimuli-sensitive polymeric materials, storage stability, and resistance toward dilution. In addition, the nanoscale sizes of polymeric micelles are distributed in an extremely confined manner. The vast majority of polymeric micelles containing hydrophobic small molecules were designed with the intention of delivering hydrophobic anticancer drugs, the administration of which normally necessitates the injection of surfactants as well as organic solvents. Micelles, because of their core-shell structure, have the ability to shield pharmaceuticals against oxidation in both in vitro and in vivo settings. It is also feasible to produce polymeric micelles by using the appropriate pharmacological chemicals [34]. 

Anticancer drugs need frequent dosing during the course of treatment to keep an effective concentration of the drug in the tumor sites. The severity of chronic toxicities and even the development of acquired drug resistance can both be a consequence of this. Therefore, polymeric micelles are highly advantageous for stabilizing the drugs in aqueous conditions, shielding the agents from the outside environment within their core, maintaining stable blood circulation, and specifically accumulating in solid tumors where they can release the loaded drugs in a controlled manner. This is because they can shield the agents from the outside environment within their core. Moreover, they are advantageous for maintaining stable blood circulation. Drugs can be physically incorporated into the core of micelles by one of the two methods: (i) by the interaction with the hydrophobic core-forming segment of the polymer, or (ii) they can be conjugated to the polymer backbone using labile bonds, which can be cleaved under specific conditions to recover the active drug [36,43,94]. 

To achieve targeted drug release, the micelles system has been modified to make it responsive to stimuli within the tumors. The nanoparticles and micelles are programmed to deteriorate or disassemble in response to the stimuli that are present at the target site or that are applied externally in order to free the payload that is inside. External stimuli include light, temperature, and localized magnetic fields, whereas TME-specific ligands include pH, upregulated enzymes, and a hypoxic environment. Due to the fact that they have shifted their metabolism away from that of normal cells, tumor cells create lactic acid as a result of their adaptation to anaerobic glycolysis [95,96]. A number of enzymes, including matrix metalloproteinases, have been found to have their activity levels increased near the tumor site. Because of the weakened blood arteries, the solid tumor’s core has a poor oxygen supply. This is because oxygen cannot get to the core. As a result, various markers of hypoxia have been found to be increased in solid tumors. The easiest way to make polymeric micelles sensitive to stimuli is to introduce linkers that are sensitive to pH, enzymes, or hypoxia between the hydrophobic core and the hydrophilic corona. This makes it so that when a stimuli-trigger is applied, the linker breaks down, causing the micelles to disassemble and release the drug inside [29,34,43,94]. The stimuli-sensitive polymers used for micelle formation are shown in Table 3. 

### 4.1. pH Sensitive Micelles

To specifically target TME with specific acidity, polymeric micelles derived from pH-sensitive block copolymers have been developed. The physical or chemical properties of segments from these micelles are sensitive to moderate shifts in pH values. Particle shrinkage or disruption can be induced by the segment, leading to rapid release kinetics from the micelles at tumor sites. These pH sensitive micelles can use slight pH changes to modify the micelles’ biodistribution and their interactions with tissues and cells. These characteristics allow encapsulated drugs to circumvent issues, including nonspecific toxicity, insufficient tumor selectivity, and the emergence of multidrug resistance in tumor cells [140,141]. If the pH of the surrounding environment changes, protons will be absorbed by the block copolymer if it contains weak acidic groups or released if it has weak basic groups. The extracellular pH of healthy tissues and blood is 7.4, whereas it is between 6.0 and 6.5 in malignant tissues. Researchers have used the endosomal and lysosomal pH disparity between healthy and cancerous tissues as a trigger for the release of chemotherapy drugs. The pH-sensitive micelles can be twisted to help release drugs under moderately acidic circumstances outside or inside the tumor cells, which improves therapeutic efficacy and reduces unwanted effects because of the presence of an ionic block or an acid-labile link [142,143]. 

**Figure 2:**
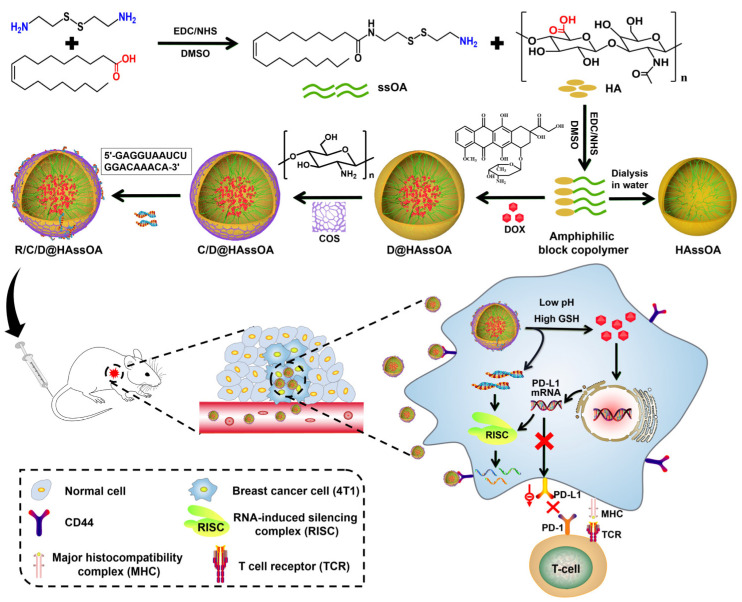
Schematic illustration of pH/redox dual-sensitive R/C/D@HAssOA synthesis and controlled release. Reprinted with permission from ref. [108]. Copyright© 2022 Elsevier B.V.

A few research reports have recently been published based on pH-sensitive micelles’ applications. Zeng et al. prepared mixed micelles from curcumin-hyaluronic acid conjugate (HC) and D-α-tocopherol acid polyethylene glycolsuccinate as carriers and dasatinib as the core. The mixed micelles were designed for co-delivery of curcumin and dasatinib for increased solubility and stability of the drugs and to increase the circulation time of micelles for an EPR effect. The system also utilized active targeting via the use of hyaluronic acid to the CD44 protein in tumor cells. The pH-sensitive ester bonds in the HC conjugate activated the micelles and release drugs in the tumor micro-acid environment. The co-delivery of curcumin and dasatinib from hyaluronic acid-based micelles effectively targeted CD4 overexpressed HepG2 cells and produced a synergistic effect. The micelles showed significant inhibition of tumor growth and reduced toxic side effects in a mouse solid tumor model of liver cancer [144]. A novel pH-sensitive drug delivery system for daunorubicin was created using poly (oligo (ethylene glycol) methyl ether methacrylate and 2-aminoethyl methacrylate hydrochloride (AMA) and 4-azibo benzyl methacrylate and 2-aminoethyl methacrylate hydrochloride. The micelles were with a particle size of 132 nm and showed 13 and 73% drug release at pH 5.0 and 7.4, respectively. The cytotoxicity against HeLa cells suggested its potential for enhanced cancer therapy. The pH-sensitive and charge-conversion micelles exhibited potential for use in cancer therapy [145]. The combination of Paclitaxel, Etoposide, and Rapamycin targets different pathways to kill cancer cells, but their low water solubility limits their clinical use. To overcome this, pH-sensitive polymeric micelles made of methyl PEG-pH-PCL polymer were developed to improve solubility and delivery to cancer cells. The pH-sensitive polymeric micelles display varying drug release behaviors based on the differential pH of tumors and healthy tissues. As the pH decreases, as in tumors, the release rate of each drug increases, resulting in improved drug levels in tumor cells. The micelles showed improved bioavailability of drugs compared to respective solutions. These drug-loaded monomethoxy PEG-pH-PCL micelles were therefore considered a beneficial option for gastric cancer treatment [146]. Lin et al. reported a pH sensitive micelle system based on O-(2-aminoethyl)-O′-methylpoly(ethylene glycol) 5000, 1-(3-aminopropyl) imidazole, and cinnamate onto polysuccinimide. The hydrophobic anticancer drug paclitaxel was successfully encapsulated within the polymeric micelles. Before being cross-linked in a low-pH environment, the drug-loaded micelles released the drug in a single burst due to the micelle-unimer transition of the polymer in the buffer solution. They demonstrated that at pH 7.4, the core cross-linked micelles released a relatively small amount of the drug, while the uncross-linked micelles released a significant amount of drug over time. This suggests that the drug circulation time can be increased, and premature drug release can be prevented by cross-linking the core of the micelles. At pH 7.4, the uncross-linked molecular state from the micelles did not provide enough protection for the paclitaxel in the core. As a result, even in physiological fluids at pH 7.4, the drugs could gradually be released from the un-cross-linked carriers before reaching the targeted cells. Drugs released too soon from uncross-linked micelle carriers in bodily fluid suggested the significance of developing micelles with cross-linked molecular states as drug carriers [147]. Song et al. developed a pH/reduction-responsive micelle for the simultaneous delivery of siPD-L1 and doxorubicin to increase the effectiveness of chemotherapy in treating cancer. The reduction-sensitive and CD44-targeting amphiphilic micelles (Hyluorpnic acid-ss-oleic acid, HAssOA) were created by joining oleic acid (OA) and hyluoronic acid (HA) with cystine. Then, doxorubicin-loaded micelles (D@HAssOA) were created by nanoprecipitating doxorubicin into the hydrophobic core of micelles. Electrostatic contact was used to coat positively charged cationic chitosan oligosaccharides (COS) on the surface of D@HAssOA in order to effectively deliver siPD-L1, as shown in Figure 2. Next, DOX and siPD-L1 co-delivering micelles (R/C/D@HAssOA) were created by electrostatic interaction loading siPD-L1. In weak acid and reduction (pH 5.0 + 10 mM GSH), doxorubicin and siPD-L1 release dramatically increased [108]. An amphiphilic triblock pH-sensitive poly(β-amino ester)-g-poly(ethylene glycol) methyl ether-cholesterol (PAE-g-MPEG-Chol) was reported to show an excellent drug release profile under different pH conditions. Doxorubicin was encapsulated into the polymeric micelles with a high drug-loading concentration. The in vitro doxorubicin release from the micelles was distinctly enhanced, with the pH decreasing from 7.4 to 6.0. Micelles exhibited excellent pH sensitivity. The release of doxorubicin was slow at a pH of 7.4. The cumulative release for the doxorubicin/polymer system was roughly 33% after 24 h, indicating that most of the drug remained in the micellar core. The doxorubicin release rate was significantly increased at pH 6.0. The drug-loaded devices showed regulated release dependent on pH change and with 35% of the drug released after 3 h and approximately 95% released after 24 h. The greater protonation of amino groups in PAE moieties under lower pH conditions may account for the loosened micelle structure. In addition, the increasing charge density on the micelle surface increased electrostatic repulsion between PAE units, which resulted in the disorganized micelle [148]. 

### 4.2. ROS Sensitive Micelles

The high level of ROS in cancer cells makes them more susceptible to damage from external ROS than normal cells that can maintain redox balance. Thus, increasing ROS production to levels greater than the hazardous threshold within cancer cells has emerged as a viable way to eliminate tumors. TME characteristics include hypoxia, moderate acidity, and elevated levels of H_2_O_2_. The presence of hypoxia in the TME facilitates tumor metastasis and increases the resistance of tumors to ROS-based cancer therapies. It has been shown that tumor oxygenation, which could significantly increase oxygen concentrations in hypoxic tumors, can help combat tumor hypoxia and make hypoxic tumors more susceptible to ROS-generated cancer therapy by exploiting the excessive buildup of ROS, particularly disease tissues [149,150,151]. In small doses, ROS, a chemical species derived from oxygen, can alter cell signaling pathways and stimulate cell growth. Increasing ROS concentration results in “oxidative stress”, since antioxidants (such as catalase or superoxide dismutase) are no longer effective in neutralizing ROS. As a result, ROS-sensitive micelles have been developed to target cancer cells specifically [25,152]. 

**Figure 3:**
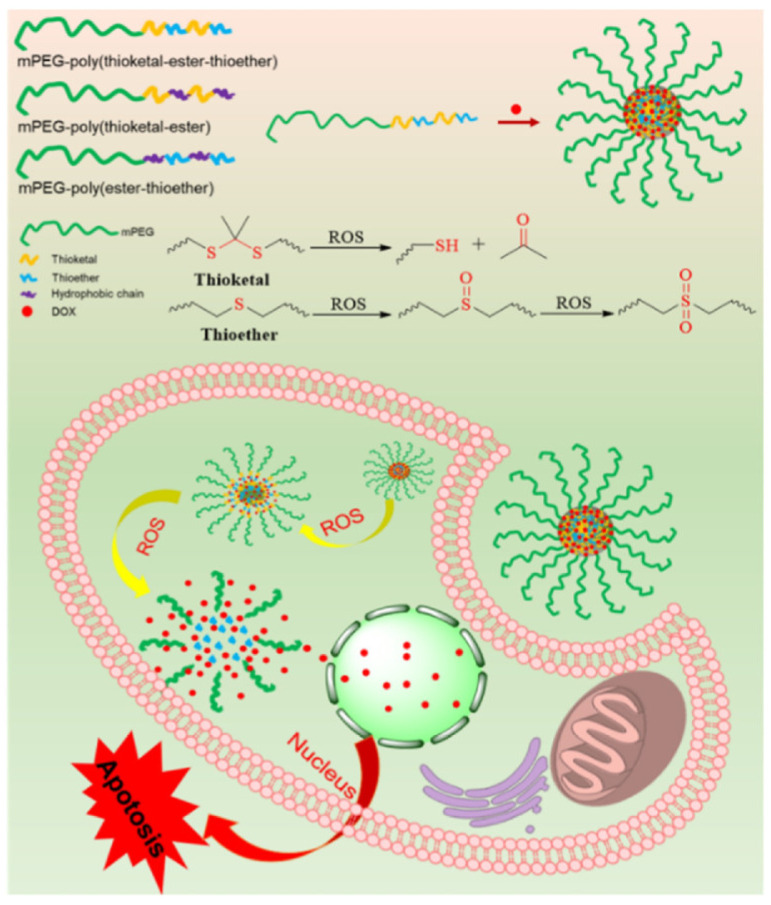
The illustration of ROS-responsive nanoparticles to induce cell apoptosis. Reprinted with permission from ref. [117]. Copyright © 2019 Elsevier B.V.

A few ROS sensitive micelles have been reported recently. For example, a dual-responsive micelle for the delivery of doxorubicin and a cyclopalladated anti-cancer agent was reported. In this micelle, the drugs were combined within the micelle’s hydrophobic core, and the micelle were decorated with an outer hydrophilic layer of PEG and β-cyclodextrin conjugate. The micelle was destabilized in response to high levels of ROS found in cancer cells or in an acidic environment, leading to the release of the drugs. The study demonstrated that the anti-cancer effects of co-delivery micelles were improved compared to free drugs in vitro [153]. Liang et al. reported the development of a ROS-responsive micelle for the co-delivery of dexamethasone and hypericin for photodynamic therapy of cancer. The micelles delivered dexamethasone to inhibit migration, invasion, and angiogenesis of vein endothelial cells, promoting the delivery of oxygen and drug-loaded micelles to the tumor site. Within the tumors, endogenous ROS partially cleaved the outer shell of the micelle to release the drugs, and an external light source was used to excite hypericin and produce ROS, leading to effective cell apoptosis. The upregulated ROS further cleaved the micelles, achieving a self-circulating burst release of hypericin and dexamethasone. This ROS-responsive platform can be used as a feasible strategy to combat cancers [154]. Sulfur-based polypeptides can show ROS-responsive structural changes, thereby providing ROS triggered release from the micellar systems. For example, a new selenium-based polypeptide with higher sensitivity to ROS-response has been developed so that they even respond to much lower levels of ROS in terms of triggered drug release. The micelles were prepared from Se-Benzyl-l-Selenocysteine N-Carboxyanhydride and methyl PEG-NH_2_. The amphiphilic copolymer was loaded with doxorubicin. These micelles selectively released their payload in tumor cells with ROS [155]. PEG biodegradable polymeric micelles with PLA, PCL, and PLGA hydrophobic blocks have been widely used as drug carriers due to their excellent biocompatibility and biodegradability [26,117,156,157,158]. Recently, a ROS-sensitive methyl poly(ethylene glycol)-poly(ester-thioether) micelles were developed and showed enhanced cellular uptake and anticancer efficacy, as shown in Figure 3. The ROS-sensitivity of the self-assembled micelles was investigated in the presence of different ROS reagents. Once the concentration of H_2_O_2_ was increased to 500 mM, the size of micelles was about 70 nm for 2 h and more than 500 nm for 4 h. It revealed that the micelles were more sensitive to high H_2_O_2_ concentrations. A similar size variation behavior of the micelles was observed in the other two ROS reagents of Fenton’s reagent and NaClO. From the size variation results, it could be concluded that the micelles were more sensitive to the ROS reagent. The methyl poly(ethylene glycol)-poly(ester-thioether) micelles showed the most efficient anticancer activity compared to methyl poly(ethylene glycol)-poly(thioketal-ester) and methyl poly(ethylene glycol)-poly(thioketal-ester-thioether) micelles [117]. 

### 4.3. Hypoxia Sensitive Micelles 

Because hypoxic cells have distinct microenvironments, a decrease in oxygen partial pressure permits tumor-specific drug delivery. Oxygen-sensitive sensors reveal that TMEs contain much less oxygen than healthy tissues. Research shows that chronic low oxygen levels alter tumor biology [94,159]. De novo angiogenesis is the process by which tumor cells generate new blood vessels in response to the inadequate blood supply. However, due to vascular hyperpermeability and accelerated permeation, these newly created blood arteries are leaky due to their discontinuous endothelium and the blockage of lymphatic drainage. Increased interstitial fluid pressure results from hypoxia-induced vascular leakage and aberrant lymphatic drainage in the tumor [160,161]. A viable technique to overcome the rising resistance to accomplish deep penetration in tumors is to progressively increase the driving force. Nitroimidazoles, nitrobenzyl alcohols, and azo linkers are representative types of hypoxia-responsive groups [16,162,163]. As a result, the hydrophobicity and surface charge of the nano-carriers undergo dramatic alterations when subjected to hypoxic circumstances. Effective drug delivery systems result from a change in hydrophilicity in response to hypoxia [162].

**Figure 4:**
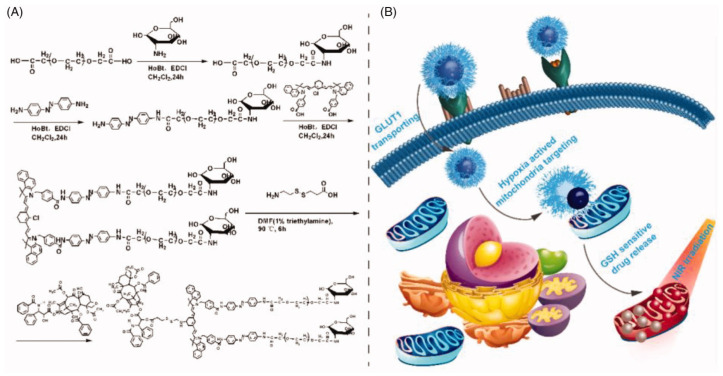
(**A**) The synthetic process of Glu-PEG-Azo-IR808-S-S-PTX conjugate; (**B**) its action mechanism after self-assembled into micelle. Abbreviations: Glu: glucose; Azo: azobenzene; PTX: Paclitaxel. Reprinted with permission from ref. [127]. Copyright 2021, published by Informa UK Limited, trading as Taylor & Francis Group.

Hypoxia sensitive micelles have shown great potentials in cancer therapy. For example, hypoxia-sensitive polymeric micelles were constructed by using a hydrophilic angelica polysaccharide, which is linked to ferrocene (using azobenzene linker), and then the side chain was covalently modified with arachidonic acid. The polymer micelles were engineered to be hypoxia-responsive and achieve selective enhancement of ferroptosis in solid tumors. In these micelles, when curcumin was incorporated, the micelles can respond to hypoxia to release drugs, and that hypoxia can enhance cell uptake and improve the proliferation inhibitory activity of HepG2 cells. This novel micellar platform has potential for the development of ferroptosis and delivery of anti-cancer drugs [164]. Mixed micelles made of folic acid and 2-(2-nitroimidazole) ethylamine conjugated poly (2-hydroxyethyl methacrylate-co-dimethylaminoethyl methacrylate-co-styrene) polymers that contain both paclitaxel and quantum dots were developed. These micelles have good drug encapsulation, storage stability, and sustained drug release properties, and exhibit enhanced cytotoxicity in MCF-7 cells and improved cellular uptake, especially under hypoxic conditions. The system also has excellent tumor targeting and hypoxia-responsive properties, and can be used for real-time in vivo imaging [165]. Recently, researchers developed hypoxia-responsive polymer micelles based on methoxyl poly(ethylene glycol)-co-poly(aspartate-nitroimidazole). The micelles were loaded with dicoumarol and sorafenib. Under low oxygen conditions, micelles cause the depletion of NADPH and inactivate quinone oxidoreductase 1, leading to the repression of hypoxia-inducible factor-1 alpha (HIF-1α). The degradation of HIF-1α increases the vulnerability of cancer cells to sorafenib-induced apoptosis, leading to increased cytotoxicity and the activation of caspase 3 and cytochrome C. The results of this study suggest that the hypoxia-responsive polymer micelles could provide a new approach for addressing hypoxia-induced drug resistance in chemotherapy [166]. Nanocarriers with positively charged surfaces have been proven in several studies to penetrate tumors more effectively. In order to overcome this problem, a hypoxia-sensitive micelle was designed, which may increase its positive surface charge in response to hypoxia gradients and therefore accomplish deep penetration in tumors. PCL served as the nanocarrier’s core, and PEG and 4-nitrobenzyl chloroformate (NBCF)-modified polylysine (PLL) formed the outer shell. During blood circulation, the NBCF-modified PLL was protected by the PEG, which provided it the capacity to block its quick removal by the immune system. Once the nanocarrier arrived at the tumor site, the hypoxic microenvironment prompted partial NBCF breakdown, which recovered the amine groups of PLL, resulting in a significant shift in the surface to be positively charged, allowing for tumor penetration. As the nanocarrier entered the core of the tumor, the decrease in oxygen content led to the further degradation of the NBCF-modified PLL, resulting in an increased positive surface charge which further promoted the deep penetration. The subsequent in vitro and in vivo investigations validated that RM/doxorubicin had a superior penetration ability and increased inhibitory efficacy on tumor tissues, which suggested its prospective applicability in cancer therapy [162]. In a recent study, a GLUT1 targeting and tumor micro-environment responsive polyprodrug-based micelle was developed, as shown in Figure 4. Amphiphilic polyprodrug conjugate glucose-PEG-aminoazobenzene-IR808-S-S-paclitaxel was constructed by using the prodrug IR808-S-S-paclitaxel as the hydrophobic block and then modifying the glycosylated PEG as the hydrophilic shell via a hypoxia sensitive linker p-aminoazobenzene. The micelles self-assembled when dissolved in water. Under the GSH reductive TME, the prodrug IR808-S-S-paclitaxel burst into paclitaxel and IR808, which then targeted tubulin and mitochondria, respectively. The micelle could be specifically transported by the GLUT1 of tumor cells and efficiently delivered paclitaxel and IR808 to the tubulin and mitochondria under the TME, ultimately leading to cell apoptosis through destroying mitochondria and depleting ATP production [127].

### 4.4. Enzyme Sensitive Micelles

The ability to selectively release their active cargo at the targeting site makes enzymatically degradable polymeric micelles promising drug delivery devices. Clearance of the delivery system is facilitated by enzymatic breakdown of the polymeric nanocarriers [167]. To transport an enzyme-sensitive substrate, these nanocarriers use the selectivity and specificity of enzymes found in cancer cells. Almost all enzyme-sensitive DDSs require extrinsic enzymes. Matrix metalloproteinases (MMPs) are the extracellular enzyme most usually employed for controlled drug discharge. Angiogenesis, invasion, metastasis, and migration are the four hallmarks of cancer, and it has been shown that MMPs play a vital role in the degradation of cell adhesion molecules. Overexpressed MMPs in the extracellular environment of many malignancies make them a promising target for drug release triggers in cancer treatment [29,94,168,169].

**Figure 5:**
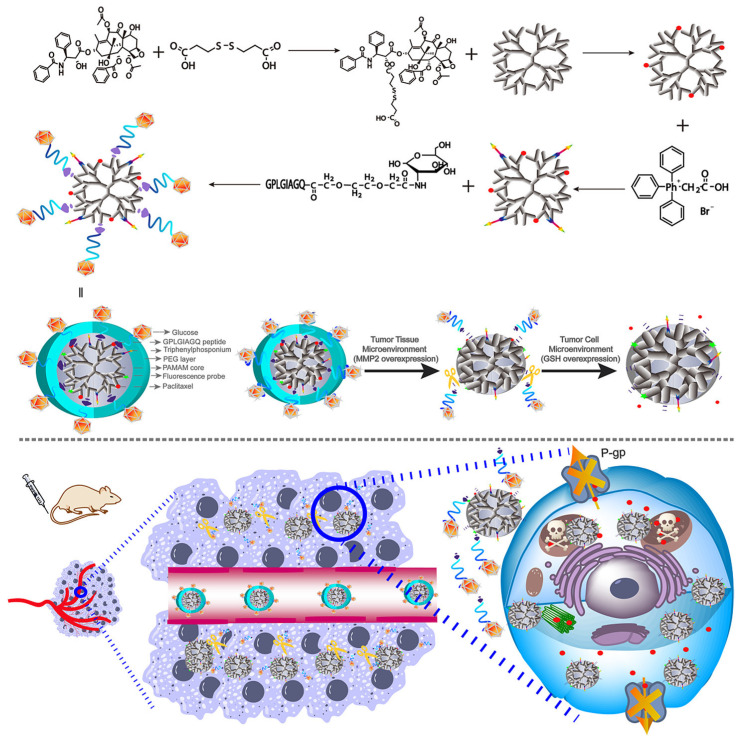
Schematic illustration of the synthesis route of the glucose-PEG–peptide–triphenylphosponium–PAMAM–PTX conjugate and its action mechanism of overcoming multidrug resistance. Abbreviation: PTX: paclitaxel. Reprinted with permission from ref. [171]. Copyright © 2018 American Chemical Society.

Han et al. constructed a polypeptide-based micellar system that is responsive to the enzyme MMP-2 for tumor immune microenvironment reprogramming. The micelles encapsulated an aryl hydrocarbon receptor inhibitor in its hydrophobic core and anti-CD28 is loaded through an MMP-sensitive peptide segment. The micelles are passively delivered to the tumor tissues and ensure the controlled release of both drugs in response to the enriched MMP-2 expression in the TME. The results of in vivo and in vitro studies show that the Dual-SHRP system performed well as a breast cancer immunotherapy, increasing the percentage of CD8+ T cells and decreasing the ratio of immunosuppressive lymphocytes within the tumor [170]. A glucose transporter-mediated and MMP2-triggered mitochondrion-targeting conjugate [glucose-PEG–peptide–triphenylphosponium–polyamidoamine-paclitaxel] composed of a polyamidoamine (PAMAM) dendrimer and enzymatic detachable glucose-PEG was constructed for mitochondrial delivery of paclitaxel, as shown in Figure 5. This conjugate was shown to target mitochondria via the glucose transporter and to be activated by MMP2. The conjugate’s sphere-shaped particles, sensitivity to MMP2, and sensitivity to GSH all play a role in the release of paclitaxel. The core of the conjugate was a PAMAM dendrimer polymer, which was co-modified with mitochondria-targeting molecular triphenylphosphine via an amido bond and model drug paclitaxel via a disulfide bond; the paclitaxel was then conjugated with the long circulating PEG layer via the MMP2-sensitive peptide. As a result, the increased EPR effect allowed the conjugates to effectively accumulate in tumor tissue. After the system binds to GLUT1 on tumor cells, the MMP2-sensitive peptide linker was disrupted, and the PEG layer separated from PAMAM. The triphenylphosphine then directed the conjugate to the mitochondria, where paclitaxel was promptly released through a reductive process into the cytoplasm and mitochondria. Overcoming tumor cell multidrug resistance required a high enough intracellular concentration of paclitaxel to block the efflux action of P-glycoprotein (P-gp) while directly acting on the mitochondria to cut off the energy source of P-gp [171].

### 4.5. Thermo Sensitive Micelles

The temperature has been identified as one of the triggers for cancer drug delivery applications. Thermosensitive synthetic polymers include PNIPAM, poly(N-vinylcaprolactam (PNVCL), poly(N-vinylisobutyramide, poly(N-vinyl-n-butyramide), poly(2-isopropyl-2-oxazoline, and poly[2-(2-ethoxy)ethoxyethoxyethyl vinyl ether]. PNIPAM, as one of the most common thermo-responsive polymers, has a LCST in an aqueous solution at 32 °C. Once the LCST has been reached, the polymer will become water soluble. Above the LCST, however, it loses its solubility in water due to a decrease in the strength of the hydrogen bonds that hold the polymer and the water together and an increase in the importance of the hydrophobic interaction [94]. In an aqueous media, pNIPAAm can undergo a phase change that can be reversed due to temperature changes [172,173,174].

Here are some recent applications of thermosensitive micelles. A thermosensitive co-polymer hydrogel system made from gelatin and Pluronic^®^ F127 was developed. This system is capable of a sustained release of a nitric oxide donor and an antibody-blocking immune checkpoint cytotoxic T-lymphocyte-associated protein-4. The unique gel formation and degradation properties of the hydrogel allow for drug retention at the tumor site and triggering release by the TME, and the formation of in situ micelles with the size enables lymphatic uptake. This platform thus represents a technology highly amenable to clinical translation to enable nitric oxide’s immune-modulatory functions to improve the therapeutic index of immune checkpoint blockade therapy [175]. Yu et al. fabricated sodium alginate-graft-poly(N-isopropylacrylamide) (SA-g-PNIPAM) biocompatible thermo-sensitive micelles by electrostatic interactions between divalent cationic metal ions and anionic SA-g-PNIPAM. The influence of temperature on the release of 5-Fluorouracil (5-FU) from SA-g-PNIPAM complex micelles was examined at below and above the LCST. At 37 °C, the drug release of the 5-FU was obviously higher than that released at 25 °C. The temperature at 25 °C was lower than the cloud-point temperature of polymeric micelles. The molecular chains of PNIPAM were stretched due to the solvation of water. It was difficult for drugs encapsulated in micelles to come out. However, when the temperature reached 37 °C, the PNIPAM chains became hydrophobic, which led to the partial dissociation of polymer micelles and accelerated the drug release. 5-FU acquired energy as the temperature increased, and, as a result, the inter-molecular forces between 5-FU and polymer micelles weakened. Based on these mechanisms, the drug release of 5-FU at 37 °C was higher than that released at 25 °C. This behavior demonstrated that the release rate of 5-FU was controlled by changing external conditions [176,177].

Han et al. reported deoxycholic acid-conjugated monomethoxy polyethylene glycol (mPEG-DC) forming thermosensitive micelles with rigid cores. The mPEG-DC thermosensitive micelles were employed to deliver estradiol to the target site of action. After 16 days, there was no noticeable increase in the estradiol release from the thermosensitive mPEG-DC micelles. The LCST behavior of the aqueous polymer solution coated the estradiol-enclosed micelles with monomethoxy polyethylene glycol shells, explaining why there was no initial burst. The LCST of the aqueous mPEG-DC solution was determined to be between 30 and 35 °C and was found to be concentration independent over the range of 0.1 to 10.0 wt.%. The DC core of the mPEG-DC micelles remained, as evidenced by the fact that it continued to exist as a collapsed peak at LCST. Furthermore, unlike with free estradiol, the release of estradiol from the micelle was diffusion-controlled rather than an explosive release at the outset. The micelle exhibited thermoreversible behavior [134].

Another heavily studied thermo-sensitive polymer is Pluronic F127, due to its exceptional thermal response and its role in regulating drug release. Pluronic F127 is made of poly(ethylene oxide), poly(propylene oxide), and poly(ethylene oxide). However, Pluronic F127’s potential applications as drug delivery systems are hampered by its unacceptably high CMC and low LCST. The modification of hydrophobic polyester blocks on Pluronic F127 is a potential solution to this issue because it improves the copolymer’s biocompatibility and biodegradability while also increasing its stability and LCST. The decreasing temperature has been reported to be effective in forming a temperature-triggered “on-off” nanocarrier in F127 [178,179,180].

**Figure 6:**
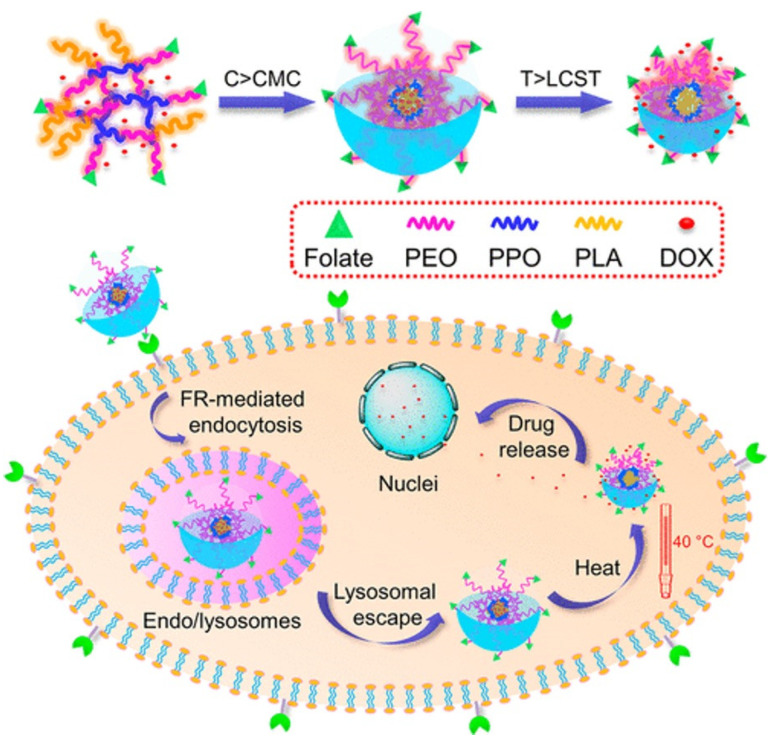
Schematic Representation of Thermosensitive Nanocarrier Working as a Targeted Drug Delivery System with Controlled Drug Release. Reprinted with permission from ref. [178]. Copyright © 2014 American Chemical Society.

Guo et al. fabricated Pluronic F127-PLA (FP) copolymer decorated with folic acid ligands-based thermo-sensitive micelles with an active targeting capacity, as shown in Figure 6. These amphiphilic copolymer-formed micelles in an aqueous solution can be taken up by FR-overexpressed tumor cells via receptor-mediated endocytosis and then rapidly released inside the cells under modest hyperthermia (40 °C). The critical solution temperature of FP100 micelles (containing a PLA segment with a polymerization degree of 100) was 39.2 °C and were suitable for use at or near body temperature. While the anticancer drug doxorubicin was released slowly from FP100 micelles at room temperature (37 °C), its concentration was rapidly increased by the shrinkage of thermo-sensitive segments at an enhanced temperature (40 °C). FP100 micelles exhibited excellent thermo sensitivity with a suitable LCST value of 39.2 °C. Under low hyperthermia (40 °C), the encapsulated anticancer drug in these micelles was rapidly released while it remained stable at 37 °C [178].

### 4.6. Magnetic Sensitive Micelles

Nanoparticles of magnesium oxide (MgO), magnetite (Fe_3_O_4_), and maghemite (Fe_3_O_3_) are widely employed to create magnetic sensitivity. It is because of their extraordinary super paramagnetism and diminutive size that they are commonly referred to as super-paramagnetic iron oxide nanoparticles. In the presence of a magnetic field, they become attracted to it, but this attraction quickly dissipates once the field is no longer there. Studies have found that controlled drug release is accomplished through two distinct mechanisms: magnetic field-induced hyperthermia and magnetic field-guided drug targeting. Hyperthermia-based magnetically induced drug release systems have been investigated throughout the past few decades [94,168,181,182].

Lin et al. reported a dual targeting method for the anti-neoplastic medicine paclitaxel using magnetic particles and an RGD peptide to achieve more cell cytotoxicity with a reduced drug dose. The amphiphilic polymer poly[(N-isopropylacrylamide-r-acrylamide)-b-L-lactic acid] (PNAL) was used as micelle-forming materials to solve the problems of water-insolubility of oleic acid-stabilized superparamagnetic iron oxide nanoparticles (SPIONs) and low incorporation efficiency of hydrophobic paclitaxel with SPION nanocarriers. The magnetic particles were then concentrated on the target cells by the influence of an external magnetic field generated by magnets. Hydrophilic PNA interacts with the aqueous environment, while hydrophobic segments form aggregates inside the micelles, where carboxylic acid stabilized-SPIONs and hydrophobic medicines can be included. PNAL-SPIONs are modified with a targeting moiety, a peptide called GGGGRGD that contains an RGD sequence and has a short linker linked to it by homo-crosslinking. The physical targeting and biochemical targeting of the micelles had a synergistic effect [138].

**Figure 7:**
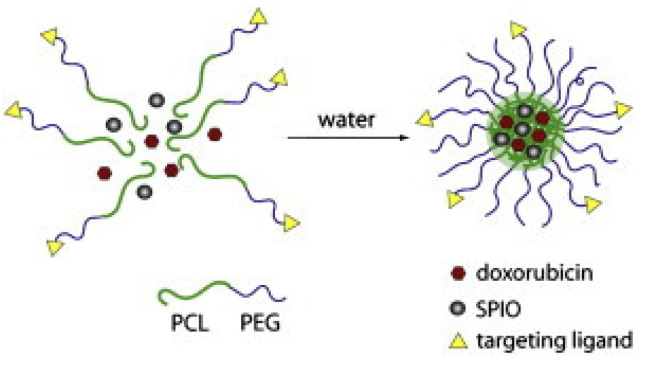
Schematic illustration of metal ions induced complex micelles for drug encapsulation. Reprinted with permission from ref. [183]. Copyright © 2018 Elsevier B.V.

Micelles based on copolymers of PCL and PEG bearing folate on the PEG distal ends, denoted as folate–PEG–PCL, were developed by Yang et al. and were used to encapsulate the anticancer drug doxorubicin and superparamagnetic iron oxide (SPIO), as shown in Figure 7. Micelles with sizes of fewer than 100 nm contained both SPIO nanoparticles and the anticancer medication doxorubicin. The micelles were superparamagnetic at ambient temperature but became ferrimagnetic at 10 K. In vitro studies showed that these polymeric micelles could serve as an efficient dual targeting nanoplatform for the delivery of anticancer medicines. Micelles functionalized with folate were recognized and taken up by tumor cells that overexpressed folate receptors; an external magnetic field improved the efficiency with which the SPIO-loaded and folate-functionalized micelles were transported into the tumor cells. The micelles showed excellent efficacy and potentials to deliver drugs and SPIO [183].

## 5. Fate of Micelles Post Administration

The fate of nanoparticles is decided by their physicochemical properties such as size, surface charge, and hydrophobicity. For example, follicle associated epithelia mediated transcellular uptake of smaller nanomaterial is higher compared to larger ones [42,184,185,186]. Cellular entry of nanoparticles through the endocytotic route include clathrin- and caveola-mediated endocytosis, pinocytosis, potocytosis, and patocytosis [187]. Contrarily, larger particles are opsonized and removed by the reticuloendothelial system (RES) related macrophagial phagocytosis. Considering the opsonization phenomenon, the nanoparticles are engineered such that the surfaces are coated with hydrophilic material, including surfactants such as polysorbate 80, hydrophilic polymers such as poloxamers, PEGs, PEO, poloxamine, etc. [188]. Surface charge and strategic functionalization can result in a transcellular transport of nanodrugs. For example, positive surface charge of the nanodrug can complex with the anionic moieties (sulfate sialic acid, sugars) of mucin resulting in enhanced transcellular transport across mucus and internalization by epithelial cells [184]. In addition, paracellular transport is enhanced in nanodrugs composed of bioadhesive polymers. The delivery of nanodrugs can be made targeted by modifying the surfaces of nanodrugs with antibodies, proteins etc. [189]. Targeted action can improve therapeutic potential and decrease toxicity. Nanotechnology-based drug delivery systems are administered either via injection, orally, or by a transdermal route [190,191,192]. Currently, the ongoing nanoparticle studies also include the pulmonary route of delivery through inhalations. In the majority of these cases, inhaled particles other than nanoparticles have limited duration of action as they undergo pulmonary clearance, including mucociliary and macrophage clearance [190,191,192]. Nanoparticles, in contrast, have long residence times as they avoid mucociliary and macrophage clearance and, thereby, enhance the therapeutic action of encapsulated active moieties [193].

Micellar nanoparticles have been proven to demonstrate a therapeutic potential for pharmaceutical drug delivery and are considered a potential alternative to liposomes. The concept of micelles has been undertaken due to their ability to solubilize the API and enhance blood circulation time, resulting in targeted delivery and enhanced therapeutic efficacy. However, the circulation time of micelles in the blood depends on biodistribution, metabolism, and clearance, which depends, in turn, on the physicochemical properties of micelles such as the size and shape, core properties, surface modifications, and surface charge as well as targeting ligand functionalization [194]. Due to the minimal surface area, micelles of ∼3–5 nm size are not cleared by macrophages and thus are excreted by the kidney. Small particles easily cross tight endothelial junctions and reach extravascular extracellular space (EES) and target organs, and thus exhibit wide distributions [194,195]. However, large-size (≥10 nm) particles are easily cleared through the liver and spleen. Small-sized polymeric micelles take advantage of the EPR effect and passively diffuse through the endothelial lining, and due to poor lymphatic drainage, they remain at the site of inflammation site and thus show targeted site-specific action [196]. Despite the stability, the leaky nature of ω-methoxy poly (ethylene glycol)-b-(N-(2-benzoyloxypropyl) methacrylamide) polymer used for curcumin delivery with more than a 70% release in plasma at 37 °C has negated the advantage of the EPR effect [197]. The same effect of curcumin leakage was also shown in other forms of delivery liposomes (180 nm) and intralipid nanoparticles (280 nm) [198].

Thus, the physicochemical properties and the effect of drug and polymeric/lipidic material and their interaction must be considered while aiming for an optimal formulation. In addition, and as discussed earlier, the size, shape, and surface charge are important for micellar biodistribution and clearance. Even though positively charged micelles are readily taken up by the RES compared to neutral and negatively charged micelles, the rate of cellular uptake and blood circulation time were found to be low in negatively charged micelles [194]. For instance, the pharmacokinetic activity of Tyr-PEG/PDLLA (neutral) and Tyr-Glu-PEG/PDLLA (anionic) micelles in mice showed that although both exhibited similar blood clearance kinetics, anionic micelles demonstrated approximately a 10 times lower biodistribution in the liver and the spleen compared to neutral micelles, due to synergic steric and electrostatic repulsion [198,199]. In addition, the composition of the core also determines the fate (biodistribution, clearance) of micelles. A study was performed to evaluate the clearance of paclitaxel-loaded pluronic P105 micelles and mixed micelles composed of similar copolymer with the addition of hydrophobic lamella [200]. The mixed micelles with additional hydrophobic lamella exhibited a significant decrease in clearance compared to just paclitaxel-loaded pluronic P105 micelles, and this can be due to the increased stability of micelles against liver uptake due to increased hydrophobic interactions in the core of micelles [200].

Following IV, administration micelles were proven to show better tissue distribution and clearance, resulting in better clinical outcomes compared to free drugs. For instance, micellar nanoparticles administered vial liquid eye drops, resulting in reduced elimination of drugs from the precorneal area compared to free drugs, and thereby increasing the therapeutic duration of action. Self-micellizing solid dispersion of tranilast using an amphiphilic block copolymer of 2-methacryloyloxyethyl phosphorylcholine (MPC) unit and a n-butyl methacrylate (BMA) unit ([poly(MPC-co-BMA)]) rapidly formed micelles of 100–150 nm diameter and showed a significant improvement in dissolution behavior [201]. Furthermore, a 50-fold enhancement of oral bioavailability and accelerate absorption of tranilast was observed in rats. In another study, the accumulation of polymeric micelles of a size of 65 nm into a subcutaneous BxPC3 tumor was observed and compared to a liposomal doxorubicin (Doxil) carrier with a size of 108 nm [201]. The results indicate that the permeability of smaller micelles into deeper tumor tissue (the region distant from blood vasculature) was higher compared to the larger liposomes, with micellar formulation showing significantly stronger anti-tumor activity than the liposome with the aid of TGF-b inhibitor [201].

## 6. Clinical Trials on Micellar Drug Delivery Systems

Many clinical studies on micellar drug delivery are in progress that aim to enhance the pharmacokinetic (PK) profiles and reduce toxicity effects in cancer therapy. The results from these studies suggest that micelles improve the pharmacokinetics and pharmacodynamics of anti-cancer drugs [202]. One such novel formulation is Genexol^®^-PM, which is a paclitaxel formulation encapsulated in polymeric micelles (PEG-PLA).

Paclitaxel on its own has low water solubility, and therefore Taxol, a solution in Cremophor EL/ethanol, was developed. However, Cremophor has been associated with several side effects, such as hypersensitivity, nephrotoxicity, neurotoxicity, neuropathy, and neutropenia [203,204,205]. Hence, micellar formulations with no Cremophor have been developed. A Genexol^®^-PM 30 mg injection is used in chemotherapy to treat various cancers, including breast cancer and ovarian cancer. It has been approved for use in Bulgaria, Hungary, and South Korea, and is being evaluated in Phase II trials in the US. The same drug is marketed under the name Cynviloq™ in other countries. Compared to Taxol^®^, Genexol^®^-PM has a high drug solubilizing capacity of about 25%, higher maximum tolerated doses, and median lethal dose, and it demonstrates linear PK behavior. Additionally, it has been shown to have higher tumor accumulation, decreased myelosuppression, and effective P-gp inhibition. Several other micellar formulations are in different stages of stage clinical trials for the treatment of various cancers (Table 4). These trials are aimed at exploring the potential of micellar drug delivery in cancer treatment. All the information of the clinical trials in Table 4 was from https://clinicaltrials.gov (accessed on 31 January 2023).

### 6.1. Paclitaxel Micellar Formulations

Paclitaxel is a taxane class drug with potential anti-cancer properties, as it blocks the breakdown of free tubulin microtubules. Taxol^®^ is a form of Paclitaxel that uses a mixture of polyoxyethylene castor oil and dehydrated alcohol, but the required doses often result in acute allergic reactions due to the non-ionic surfactant [206,207]. Genexol-PM, made by Samyang Co. in Seoul, is a less toxic version of Paclitaxel, formulated as a 25-nm diameter micellar complex of PEG and PLA. It was produced by dissolving the block copolymer and drug in acetonitrile, evaporating the solvent, and then dissolving the resulting gel in preheated water to form paclitaxel-filled micelles [208]. Genexol-PM is approved for the treatment of breast, lung, and ovarian cancers, and is undergoing clinical evaluation for use in other cancers (Table 4). Clinical studies of Genexol-PM in pancreatic and urothelial cancers showed that the drug was well tolerated and showed good antitumor activity [209].

The NK105 drug delivery system (Nippon Kayaku Co., Tokyo, Japan.) is a polymeric micelle na-noparticle that encapsulates paclitaxel. It has a diameter of about 85 nm and is comprised of an amphiphilic copolymer made up of a hydrophilic block of PEG and a hydrophobic block of polyaspartate modified with 4-phenyl-1-butanol. The copolymer is designed to create a microenvironment within the micelle core that enables high drug loading and retention, with the goal of producing a drug carrier that retains the drug after intravenous administration. This first-generation technology results in a prolonged half-life of the drug (more than 10 h) compared to Taxol (30 min), and the second-generation nano-formulations in clinical development include drugs that are covalently conjugated or chelated to the core-forming block of the micelles [210,211]. It was developed as a safer alternative to traditional paclitaxel formulations, which are solubilized in ethanol or polyoxyethylene castor oil and can cause serious hypersensitivity reactions and anaphylactic shock. In vivo studies in mice showed that the Cmax and AUC were increased 3- and 25-fold, respectively, with NK105 compared to free paclitaxel [210]. A Phase II clinical study with NK105 showed a 15-fold higher AUC than conventional paclitaxel. The efficacy of NK105 has been attributed to its EPR, a unique phenomenon in solid tumor tissue, and previous clinical studies have shown its potential––with a good response rate in advanced gastric cancer, and with a partial response or stable disease in solid tumors and breast cancer [212,213]. In a Phase III trial, NK105 (a nanoparticle drug delivery formulation of paclitaxel) was compared to paclitaxel for the treatment of metastatic or recurrent breast cancer. Both treatments were given to patients in a 28-day cycle, with NK105 given at 65 mg/m^2^ and paclitaxel at 80 mg/m^2^. The primary endpoint was progression-free survival, but the results showed that both treatments had similar efficacy with a median progression-free survival of 8.4 and 8.5 months for NK105 and paclitaxel, respectively. The safety profile of the two treatments was similar, but the incidence of peripheral sensory neuropathy was lower in patients receiving NK105. The patient-reported outcomes for peripheral sensory neuropathy were significantly better in the NK105 group [214]. The Phase III studies on NK105 resulted in a failure to meet endpoints. Nippon Kayaku Co. stated that the study’s main objective, which was progression-free survival, failed to meet its pre-determined statistical standards. However, the drug NK105 was well-tolerated compared to paclitaxel. Currently the paclitaxel micellar formulations, Cynvilog (polymer-based) Paclical polymer-based), are approved in South Korea and Russia, respectively [211].

Nanoxel-PM is a docetaxel-loaded PEG2000-PDLLA1765 micellar formulation with 25 nm sized particles (Samyang company, Seoul, Korea). The formulation was created by dissolving docetaxel and copolymer in ethanol, evaporating the solvent, and then dispersing the matrix in water. D-mannitol was added as a cryoprotectant. The bioequivalence of Nanoxel-PM to Taxotere^®^ was determined to be within 100% ± 20% which were performed on based on mice, rats, and beagle dogs. Clinical trials for Nanoxel-PM have been registered but no results have been published [215,216].

Paccal Vet is a new form of PTX combined with a surfactant-based derivative of retinoic acid for treatment of mast cell tumors in dogs. In a study of 29 dogs, 59% showed complete or partial responses with a median progression-free survival of 247 days. A Phase III study showed Paccal Vet to be clinically safe and effective with better response rates than lomustine, and fewer adverse effects. Paccal Vet has been granted MUMS status and approved by the FDA for use in certain types of dog cancer but can only be used for the approved indications [209].

Triolimus (Co-D Therapeutics) is a polymeric micelle drug delivery system that contains a combination of three drugs: paclitaxel, rapamycin (mTOR inhibitor), and tanespimycin (Hsp90 inhibitor). It is a 30–40 nm diameter PEG-b-PLA micelle formulation. Triolimus has received orphan drug designation for angiosarcoma and is in late-stage preclinical evaluation for the treatment of breast cancer, non-small cell lung cancer, and angiosarcoma. The use of a polymeric nanomedicine delivering multiple drugs is close to entering the market through clinical trials [217,218].

### 6.2. SN38 (Irinotecan Metabolite (NK012) Micelles

NK012 is a polymeric micelle formulation of SN-38, the active metabolite of irinotecan [219]. NK012 releases its active ingredient via hydrolysis and does not require metabolic conversion by enzymes. This makes it a promising agent for clinical use, as its ability to suppress tumor growth and its antitumor effects in various cell types, including human tumors, are stronger than those of the active metabolite. In addition, NK012 accumulates in high concentrations in tumors and has been shown to result in less severe diarrhea compared to the active metabolite in preclinical studies [220]. A clinical study evaluated the efficacy and safety of NK012 in Japanese patients with unresectable metastatic colorectal cancer through a multicenter open-label Phase II trial. The study consisted of 58 patients divided into two groups, with group A as the primary efficacy population. The primary endpoint was the response rate, which was 3.8% in group A, with median progression-free survival and overall survival of 3.30 months and 15.03 months, respectively. The most common adverse drug reaction was neutropenia, while the incidence of grade three diarrhea was low or zero. No treatment-related deaths were reported. The study concluded that the response rate of NK012 monotherapy was similar to that of irinotecan monotherapy reported in a Phase III trial, but the initial dose of 28 mg/m^2^ may be too high for colorectal cancer patients who have previously been treated with oxaliplatin-based chemotherapy [221]. NK012 is a polymeric micelle formulation of SN-38, the active metabolite of irinotecan [200].

### 6.3. Anthracycline Class Drugs—Micellar Formulations

The NK911 is a micellar formulation of doxorubicin that is composed of a copolymer of PEG and polyaspartic acid developed in Japan. Doxorubicin is partially attached to the side chains of aspartic acid to increase its hydrophobicity, resulting in a hydrophobic core within the micelle. This allows for the encapsulation of free doxorubicin, making the loaded drug responsible for its antitumor activity [222]. The NK911 micelles have a small size of around 40 nm and have been shown to accumulate in solid tumors in mice, leading to a Phase I clinical trial with 23 patients with metastatic or recurrent solid tumors. The trial aimed to study the pharmacokinetic profile of the nanotherapeutic through the maximum tolerated dose and toxicity, with administration starting at 6 mg/m^2^ doxorubicin equivalent every 3 weeks. The NK911 micelles showed an increased plasma half-life and plasma concentrations of the drug compared to free doxorubicin but had lower stability in plasma compared to liposomal doxorubicin. The most common side effect was neutropenia, but the treatment was generally well tolerated and had a good safety profile [223]. A Phase II clinical trial was proposed with a recommended dose of 50 mg/m^2^ every 3 weeks, but it is unclear if it proceeded.

Sp1049C, developed by Supratek Pharma Inc. in Canada, is a 30 nm diameter mi-cellar formulation that contains doxorubicin (8.2%) loaded into a blend of two Pluronic block copolymers (Pluronic F127 and Pluronic L61 with a ratio of 1:8). Pluronic F127 serves to stabilize the micelles while Pluronic L61 enhances the effectiveness of the treatment by inhibiting P-gp efflux transporter [224]. In a Phase I clinical trial, this formulation was evaluated in 26 patients with advanced solid tumors. The trial administered doses ranging from 5 to 90 mg/m^2^ every 3 weeks for at least six cycles. The pharmacokinetics showed linearity and a longer half-life than conventional doxorubicin. The maximum tolerated dose was 70 mg/m^2^, and side effects included myelosuppression, alopecia, stomatitis, and transient lethargy. No patients showed a complete or partial response, but 30.8% had stable disease for a median time of 17.5 weeks [225]. A Phase II trial showed notable single-agent activity and an acceptable safety profile in 21 patients with advanced adenocarcinoma of the esophagus and gastroesophageal junction. The objective response rate was 47%, median overall survival was 10 months, and median progression-free survival was 6.6 months. The predominant toxicity was neutropenia [226]. A Phase III trial for gastrointestinal cancer was conducted but no results have been published.

NC-6300/K-912 is a pro-drug conjugate made by linking the chemotherapy drug epirubicin to PEG-poly(α,β-aspartic acid) through an acid-sensitive hydrazone bond. This results in a micellar formulation with a particle size of 40–80 nm and selectively accumulates in tumor tissue due to the EPR effect and the release of the drug in acidic tumor environments. In a Phase I clinical trial, 19 patients with advanced or recurrent solid tumors were given doses of NC-6300/K-912 to determine safety, recommended dosage, tolerability, and pharmacokinetics. The maximum tolerated dose was found to be 170 mg/m^2^ and 1 patient out of 19 had a partial response, with an objective response rate of 5%. The trial showed that NC-6300/K-912 was well tolerated, with lower toxicity compared to conventional epirubicin [227,228].

### 6.4. NC-4016/Oxaliplatin Micelles

NC-4016 is a PEG-b-poly (L-glutamic acid) copolymer-based micelle of size 40 nm developed to deliver oxaliplatin for advanced solid tumor treatment to decrease drug-related toxicity. Oxaliplatin is incorporated into the micelles in its active metabolite form dichloro (1,2-diamino cyclohexane) platinum(II) (DACHPt) [229]. In vivo studies in a mouse model demonstrated that the antitumor effects of NC-4016 are comparable to oxaliplatin. Other animal studies have also demonstrated higher efficacy in tumor models, including human pancreatic, murine colon carcinoma, and melanoma [230]. Compared to oxaliplatin alone, oxaliplatin loaded micelles showed enhanced stability in physiological conditions, with extended blood circulation time and with more than a 1000-fold increase in plasma drug concentration from 0 to 72 h.

### 6.5. NC-6004/Cisplatin Micelles

NC-6004 (NanoplatinTM) is a PEG-b-poly (l-glutamic acid) based polymeric micelle formulation for cisplatin delivery with a micelle size ~28 nm [231]. NC-6004 is composed of micelles of size ~30 nm. NC-6004 is made by reacting the sodium salt of PEG-P(Glu) and Cisplatin in water to create Cisplatin-incorporating micelles. Cisplatin is released from NC-6004 in the presence of chloride ions through an exchange reaction between the carboxylic groups in P(Glu) and chloride ions. A micelle-based formulation was developed to alter the bio-distribution and PK profile, thereby increasing the tumor accumulation and efficacy [232].

A Phase I clinical trial of NC-6004 for advanced solid tumors was conducted in the UK, with doses ranging from 10 to 120 mg/m^2^. The maximum tolerated dose (MTD) was determined to be 120 mg/m^2^ due to renal impairment and hypersensitivity reactions. The recommended dose for Phase II was 90 mg/m^2^. There was no complete or partial response in patients, with 41.2% of patients having stable disease. The median progression-free survival was 49 days, and 82.4% of patients died or had tumor progression. A Phase I clinical trial of NC-6004 in combination with gemcitabine for advanced solid tumors was conducted in Japan. The most common side effects were decreases in neutrophil and white blood cell count. The MTD was determined to be 90 mg/m^2^, and the recommended dose for Phase II was 60 mg/m^2^ [233]. In addition, another Phase I/II study was performed in Taiwan and Singapore on 19 patients who had pancreatic cancer and were treated with a combination therapy of NC-6004 and gemcitabine. This study also recommends a Phase II dose for the combination therapy to be 90 mg/m^2^, and the results indicated good activity and tolerability [234]. A combination Therapy With NC-6004 and Gemcitabine in advanced solid tumors or non-small cell lung, biliary, and bladder Cancer (NanoCarrier Co., Ltd., Chiba, Japan.) was submitted for clinical trials with a dose escalation, but no results were reported [235].

### 6.6. BIND-014/Docetaxel Micelles

BIND-0 Docetaxel is a taxoid that is derived from the European yew and is more potent than paclitaxel as a microtubule depolymerization inhibitor. It is used in the treatment of various cancers, including breast, lung, ovarian, and gastric cancers. However, the clinical formulation of docetaxel, Taxotere, can cause adverse effects, such as hypersensitivity reactions, hemolysis, and peripheral neuropathy [236]. BIND-014 is a nanoparticle composed of PEG-PLA, decorated with a prostate-specific membrane antigen inhibitor and encapsulating docetaxel [237]. A Phase I study of BIND-014 showed it was well-tolerated with predictable and manageable toxicity [238]. A Phase II study of BIND-014 in combination with prednisone in patients with metastatic prostate cancer showed an overall response rate of 32% and a median progression-free survival of 9.9 months [239]. Another Phase II study of BIND-014 as second-line therapy in patients with Stage III/IV non-small cell lung cancer showed a disease control rate of 63% in patients with KRAS mutations [240]. However, BIND-014 was not effective in later trials against cervical and head-and-neck cancers, leading the company to discontinue its development in 2016 [241].

## 7. Regulatory Submissions

**Figure 8:**
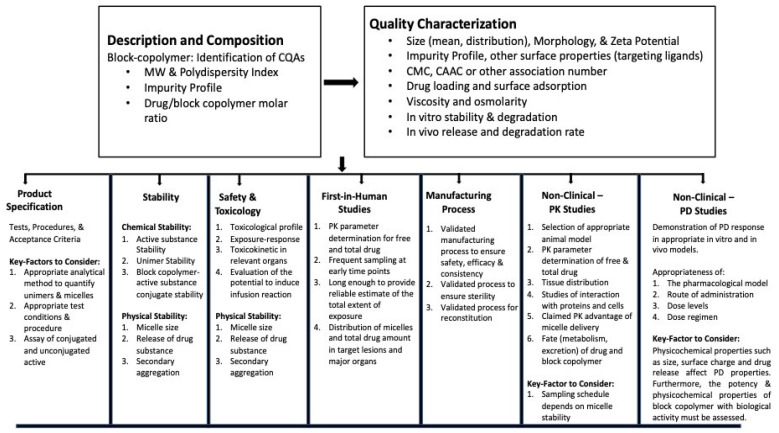
Schematic of regulatory process of a nanoparticle (including micellar nanoparticles) based drug delivery system. Abbreviations: CQA: critical quality arrtribute; MW: molecular weight; CMC: Critical Micelle Concentration; CAAC: Critical Assembly/Aggregate Concentration; PK: pharmacokinetics; PD: pharmacodynamics.

A schematic showing the regulatory process of a nanoparticle (including micellar nanoparticles) based drug delivery system is shown in Figure 8. Several clinical trials are in progress to investigate the safety and efficacy of micelles, but their scalability remains an issue to transfer basic research to clinical practice to commercialization. In addition, a lack of detailed understanding of the PK/pharmacodynamics (PD) aspects, clearance rate, and in vivo degradation profiles of the materials and micelles needs extensive research. Thus far, most of the IVIVC profiles are only confined to lethal dose (LD50), inhibitory concentration (IC50), and maximum tolerated dose (MTD). However, to obtain a complete toxicity profile, an inclusion of acute and sub-acute models, such as genotoxicity and gene expression pattern determination, must be considered. Additionally, due to the toxicity resulting from the size and altered physicochemical properties, due to the type of polymers used in the micelle construction, a separate registration requirement might be needed for micelles [242,243,244].

ICH Pharmaceutical Development Q8(R2) guideline encompasses the regulation of the pharmaceutical development of any novel formulation. Similar to other regulated products, such as a drug, device, or biologic, the Food and Drug Administration (FDA) is the regulatory authority for the approval of nanomedicines in humans in the USA [244]. However, the nanoscale size of micelles requires rigorous characterization and testing to determine the safety and efficacy profiles in humans. Extensive assessment and characterization of micelles is addressed in an EMA reflection paper published in 2013 [244,245]. The paper describes various aspects of product development, including its characterization, specifications, stability, non-clinical pharmacokinetic and pharmacodynamic aspects, and first-in-human studies. Furthermore, the paper also discusses the physicochemical characterization of actives and polymers and their chemical stability with an impurity profile. Physical characterization includes CMC, micelle size, and surface charge, micelle shape and morphology, micellar stability in plasma, and drug release in biorelevant conditions. To summarize, the key highlights of the reflection paper include micelle characterization as a representative of product specifications for its physicochemical stability, pharmacokinetics and pharmacokinetics, and toxicology studies. To exemplify, considering the stability of micelles and the fate (site and release) of the micelles and drug, PK and PD studies should be designed accordingly [244,245].

Due to the absence of compendial methods for the integrity of micelles and their release rate, in-house methods must be set up and validated accordingly to ensure repeatability and reproducibility. Methods should be developed such that the method should be able to predict the release both in the circulation and the targeted site of action to mimic the physiological environment of micelle. In addition, the method should be sensitive enough to verify batch-to-batch variability. In vitro–in vivo correlation (IVIVC) for micelles is not always straightforward, as the predictivity of in vitro tests is a critical issue. Only a few research articles have tried to address IVIVC by establishing a correlation between in vitro drug release and drug pharmacokinetics. For example, Zhang et al. established IVIVC for PEG-PCL micelles, where they found that 70% of micelles were intact in an in vitro experiment versus the 60% that were found to be in bloodstream in vivo after 72 h [246,247].

In addition, Liu et al. demonstrated the stability profiles of micelles in the bloodstream in vivo correlated with in vitro stability by employing AF4 coupled with fluorescence and DLS, which was able to study interactions between micelles and different plasma proteins. However, this concept cannot be generalized, as evident from the work published by Bagheri et al. using similar techniques to determine the fate of curcumin-loaded polymeric micelles. Although the data indicated an agreement between micelle circulation time and in vitro data in plasma using AF4, curcumin clearance was faster in vivo than in vitro, which was then attributed to curcumin/blood cell interaction [247]. These results suggest that the selected polymers, drugs, and their interaction with the blood and plasma components in vivo can have a significant impact on the method predictivity. Owing to these issues, it is recommended to set up a method considering the physicochemical interaction of the drug product in vivo, and the method should always be drug and formulation specific.

In summary, stability, extensive physicochemical characterization, and the development and validation of specific methods constitute the main barriers to micellar clinical development. Thus, it is recommended to address these concerns in order to reduce the gap between preliminary research and the progression toward commercialization.

## 8. Conclusions and Future Outlooks

Polymeric micelles have gained attention as a promising nanocarrier for drug delivery because of their advantageous properties. The biocompatibility and low toxicity of micelles improve their circulation in the body, and they can solubilize hydrophobic drugs, making them more effective for treating various diseases.

In this review, different aspects of micelle development and application were discussed, including the selection of polymers, the responsive release of drugs, the fate of micelle administration in the body, regulatory considerations, and clinical trials.

The micellar formulations for cancer treatment are very promising. One of the key advantages of micelles is their ability to target specific cancer cells and improve the intratumor concentrations with increased potency of anti-cancer drugs. Micelles can be designed to target cancer cells by using specific targeting moieties, such as antibodies or peptides, which can enhance the selectivity and specificity of drug delivery.

In addition, micelles have the potential to overcome cancer drug resistance, which is a major challenge in the treatment of many cancers. The drugs loaded within the protective micelle structure are protected or shielded from efflux pumps and other mechanisms responsible for developing drug resistance. Micelles also offer combination drug therapies by serving as a co-delivery vehicle. The stimuli-responsive polymers can also release drugs in response to specific triggers, such as changes in pH or temperature, which could improve the accuracy and effectiveness of drug delivery to cancer cells.

Micelles have shown considerable success at the clinical level. Several micelles have been approved by FDA and become commercially available. The clinical translation of micelles has seen major progress in recent years. For example, Genexol^®^-PM and NANOX-EL-M are examples of commercially available micelles mentioned in this review. However, a few obstacles still exist in micelle application. Despite their versatility, micelles still face challenges, such as delivering low payloads to target sites and ensuring clinical safety. However, researchers are actively working to overcome these challenges by designing copolymers for micelles and studying their behavior in vivo. By further developing novel copolymers used for micelles and investigating micelle in vivo kinetics, these obstacles may be overcome, and more effective therapies for a wide range of diseases may be developed.

## Figures and Tables

**Table 1:** Features of hydrophilic polymers commonly used for polymeric micelles.

Polymer	Structure	Advantages	Disadvantages	Ref
PEG	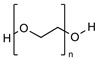	Clinically approved Stealth behavior Prolonged blood circulation Diminished RES uptake Enhanced permeability and retention effect	Unexpected changes in PK behavior Non-biodegradable	[57,58]
Polysaccharides	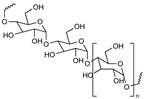	Non-toxic Biodegradable Stealth behavior Facilitating mucoadhesion Enhanced targeting of specific tissues Enhanced a reduction in the inflammatory response Easy for modification	Its degradation (oxidation) characteristics at high temperatures (above their melting point), which are often required in industrial processes Toxicity due to impurities	[59,60,61,62,63]
pHPMA	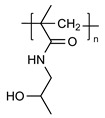	Non-toxic Non-immunogenic Biocompatible Pendant groups readily engineered	Only a few soluble drug conjugates have entered clinical trials Complicated synthesis The unsatisfactory characteristics of the conjugate molecules The tendency for such conjugates to perform differently in preclinical animal models than in the human body	[65,66,67,68,75]
Poly(acrylic acid)	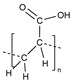	pH sensitive, mucoadhesive Biodegradable Biocompatible	Poor mechanical properties, its structures need to be modified for use	[69,70,71,76]
Poly(glutamic acid)	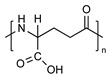	pH sensitive Biodegradable Biocompatible Easy for chemical modification	α- poly(glutamic acid) synthetically produced has a lower molecular mass which limits its application High cost of production	[71,77]
Polyvinyl alcohol	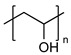	Widely used for cross-linking synthesis Biocompatible Non-immunogenic Non-toxic	Under wet conditions, its properties are diminished because of the plasticizing action of water molecules	[78,79,80]
Poly(N-vinyl-2-pyrrolidone)	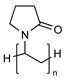	Containing cationic groups for modification	Non-biodegradable Hygroscopic	[81,82,83]
Poly(N-isopropyl acrylamide) (PNIAAm)	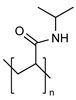	Thermo sensitive controlling payload release	In vivo studies are still pending for most copolymers, grafted polymers, and biopolymer-conjugates investigated up to today due to high cost and ethical restrictions for in vivo analysis to test their viability	[84,85]
Poly(ethylene imine)	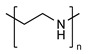	Facilitating to escape from endosome and payload release in cytoplasm Facilitating cellular uptake	Positively charged with toxicity Difficult to release negatively charged payloads due to strong electro attraction	[86]

**Table 2:** Features of hydrophobic polymers commonly used for polymeric micelles.

Polymer	Structure	Advantage	Disadvantage	Ref
Poly(histidine) (PHIS)	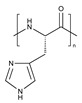	hydrophilic at acidic pH condition; hydrophobic at pH around 7.4, pH sensitive Biocompatible Biodegradable Facilitating to escape from endosome and payload release in cytoplasm	Poly(histidine) is too sensitive to environmental pH, which could affect the stability of the core The chain length also affects the anticancer efficacy and the pH responsive drug release rate	[87,88]
Polyethers (i.e., poly(propylene oxide; block copolymers such as Pluronics)	Pluronics: 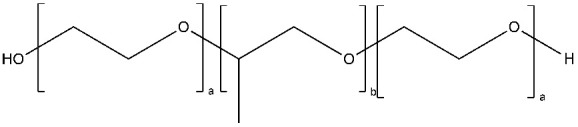	Widely used, commercially available Non-expensive Pluronics are thermoresponsive.	Low affinity with drug molecules	[34,72]
Polyesters (i.e., poly(lactide), poly(lactide-co-glycolide), poly(ε-caprolactone), poly(β-amino ester)), poly(glycolic acid), poly(lactide-co-caprolactone))	Poly(lactide) (PLA): 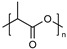 Poly(lactide-co-glycolide) (PLGA): 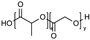 Poly(ε-caprolactone) (PCL): 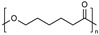 Poly(glycolic acid) (PGA): 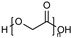 Poly(lactide-co-caprolactone) (PLCA): 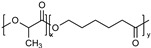	Biodegradable and biocompatible. These are the most used polymers for drug delivery. They show excellent control on the drug release rates. Poly(β-amino ester) is pH sensitive, providing stimuli-responsive drug release, and is used for gene carriers and increased cell uptake due to positive charges. Poly(glycolic acid) is thermoplastic, enabling a stimuli response.	High hydrophobicity and subsequently entrapment by macrophages through the opsonization process, long-term degradation time, and low loading for hydrophilic drugs. PLA induces the production of lactic acid due to polymer degradation, which leads to the formation of an acidic microenvironment. PLA shows initial burst release with significant drug loss and drug-related toxicity. PCL has a slow degradation time. Poly(β-amino ester): cationic charges may be toxic to cells. Poly(glycolic acid) has a fast degradation time with fast drug release.	[34,73,89,90,91,92,93]

**Table 3:** Stimuli polymeric micelles for drug delivery.

Stimuli	Polymeric Carrier	Payload	Release Mechanism	Application	Ref
pH	Poly(L-histidine)-b-poly(ethylene glycol)/poly(L-lactic acid)-b-poly(ethylene glycol-folate	Doxorubicin	Protonation of PHIS	PHIS destabilizes micelles and triggers doxorubicin release	[97]
	Poly(ethylene glycol-block-poly[(1,4-butanediol)-diacrylate-β-5-amino-1-pentanol]/2,3-dimethylmaleic anhydride-polyethyleneimine-b-poly[(1,4-butanediol)-diacrylate-β-5-amino-1-pentanol]	Paclitaxel	Protonation of poly[(1,4-butanediol)-diacrylate-β-5-amino-1-pentanol]	2,3-dimethylmaleic anhydride enhances micelles internalization; poly[(1,4-butanediol)-diacrylate-β-5- amino-1-pentanol] dissociates micelles and triggers paclitaxel release	[98]
	Methyl poly(ethylene glycol) ether-b-poly(β-amino esters)-b-poly lactic acid	Doxorubicin	Protonation of poly(β-amino esters)	Poly(β-amino esters decreases hydrophobicity of micelles at acidic condition and triggers doxorubicin release	[99]
	Poly(ethylene glycol)-poly(L-histidine)-poly(L-lactide)	Doxorubicin	Protonation of PHIS	PHIS swells and relocates to the surface of the micelles to trigger doxorubicin release	[100]
	Methoxy-poly (ethylene glycol)-b-poly (ε-caprolactone)-b-poly (diethylaminoethyl methacrylate)	Curcumin	Protonation of poly (diethylaminoethyl methacrylate)	Poly (diethylaminoethyl methacrylate) switch from hydrophobic to hydrophilic to change micelles structure and triggers release Curcumin	[101]
	Poly(2-(diisopropylamino)ethyl methacrylate-co-2-(2′,3′,5′-triiodobenzoyl)ethyl methacrylate)	Dextran/Doxorubicin	Protonation of poly(2-(diisopropylamino)	Poly(2-(diisopropylamino) switch from a hydrophobic to a hydrophilic state under acidic conditions upon protonation, which deceases the stability of micelles and triggers drug release	[102]
	Methyl ether poly(ethylene glycol)-poly(β-amino ester)	Camptothecin	Protonation of poly(β-amino ester	Poly(β-amino ester facilitates a pH-dependant micellization/demicellization transition and triggers camptothecin	[103]
	Poly (ethylene glycol) methyl ether-b-(poly lactic acid-co-poly (β-amino esters))	Doxorubicin	Protonation of poly(β-amino ester)	Poly(β-amino ester) destabilizes micelles and triggers doxorubicin release	[104]
	Methoxy poly (ethylene oxide)-b-poly (aspartate-hydrazide)	Doxorubicin/SN-38	Hydrolisis of Hydrazone bond	Se-Se bond exerting redox responsiveness and Hydrazone bond hydrolyzing decrease micelles stability and trigger Doxorubicin/SN-38 release	[105]
	Hyaluronic acid-S-S-Podophyllotoxin	Podophyllotoxin	Cleavage of acid-sensitive ester bonds	Ester bonds and disulfide bonds cleave to decrease micelle stability and podophyllotoxin releases from micelles	[106]
	Hydrazide functionalized methoxy poly(ethylene glycol)-block-poly(ε-caprolactone)	LCA	Electrorepulsion between LCA and the copolymer	Loss of ionic interaction between LCA and micelles triggers LCA release	[107]
	Chitosan coated hyaluronic acid-oleic acid	Doxorubicin/siPD-L1	Protonation of the amino group of COS	Decomposition of copolymer shell, the swelling of COS, and disulfide bond cleavage trigger drug release	[108]
	Methoxypolyethylene glycols-b-poly (6-O-methacryloyl-d-galactopyranose)-disulfide bond-doxorubicin	Doxorubicin	Destability of hydrazone bonds	The destability of hydrazone bonds decrease micelles stability; the break of disulfide bonds causes decreased hydrophobicity in the micellar inner cores and dissociates the conjugates to release doxorubicin	[109]
ROS	Polyethylene glycol-p(2-aminoethyl methacrylate hydrochloride-camptothecin conjugated hydroxyethyl methacrylate-oxalyl chloride	β-Lapachone/camptothecin	Breaking the H_2_O_2_-cleavable linkage from camptothecin	The removal of camptothecin enhances the disassembly of the micelles and drug release	[110]
	Poly(β-thioether ester)-poly (ethylene glycol)-lipoic acid	Doxorubicin	Thioether group and disulfide bond cleavage	The cleavage of disulfide bonds and β-thiopropionate linkers decrease in core crosslinking density and trigger doxorubicin release	[111]
	Methoxy poly(ethylene glycol)-thioketal-poly(ε-caprolactone)	Doxorubicin	Thioketal bond cleavage	π–π interactions increase drug loading; thioketal bond cleavage increases doxorubicin release	[112]
	Poly(l-methionine-block-l-lysine)-PLGLAG-methoxy poly(ethylene glycol)	Doxorubicin	MMP-sensitive linkers (PLGLAG) cleavage	Poly-l-lysine chains assist the cellular penetration by electrostatic interactions; thioether converts to a sulfoxide moiety to cause a phase transitions and micelle structure break to release Doxorubicin	[113]
	CD147-Carboxymethyl chitosan-phenylboronic acid pinacol ester	Doxorubicin/CD147	Oxidation of phenylboronic acid pinacol ester	The micelles exert CD147 targeting effect; ROS depolymerizes micelles and triggers doxorubicin release	[114]
	Poly(ethylene glycol)–poly[aspartamidoethyl(p-boronobenzyl)diethylammonium bromide]	miR-34a mimic/volasertib (BI6727)	Boronic acid reaction	Boronic acid produces tertiary amines and p-quinone methide to enhance micelle degradation and release drugs	[115]
	Poly(propylene sulfide)-poly(N-isopropylacrylamide)	Doxorubicin	Hydrophobic (thioether)-to-hydrophilic (sulfoxide, sulfone) transition of thioether	Poly(propylene sulfide) decreases micelles stability and triggers doxorubicin release	[116]
	Methyl ether poly(ethylene glycol)-poly(ester-thioether)	Doxorubicin	Oxidation of thioether	Enhance drug loading content via the π-π interaction	[117]
	Poly(ethylene glycol)-poly(N6-carbobenzyloxy-l-lysine)-poly(β-benzyl-l-aspartate)	Doxorubicin	Thioketal bond cleavage	The primary-amine-rich pLys block would provide interlace sites for the ROS cleavable cross-linker and then increases doxorubicin release	[118]
	Imidazole groups conjugate polyethylene glycol-conjugated triphenylphosphonium	Doxorubicin	TK bonds cleavage	Imidazole groups protonation and TK bonds cleavage release doxorubicin	[119]
Hypoxia	Poly(ethylene glycol)-*block*-poly(methacrylic acid-*co*-2-nitroimidazole methacrylate)	Doxorubicin	2-nitroimidazole converting to hydrophilic 2-aminoimidazole	2-nitroimidazole groups enhances expansion and self-disassembly of micelles, then triggers doxorubicin release	[120]
	Polyethyleneimine-C6-2-nitroimidazole	siRNA	2-nitroimidazole converting to hydrophilic 2-aminoimidazole	2-nitroimidazole elicits a loose structure to facilitate the siRNA dissociation in the cytoplasm	[121]
	Poly(ethylene glycol-poly(ε-(4-nitro)benzyloxycarbonyl-l-lysine)	Doxorubicin	Degradation of poly(ε-(4-nitro)benzyloxycarbonyl-l-lysine)	Self-immolation of poly(ε-(4-nitro)benzyloxycarbonyl-l-lysine) derivative triggers doxorubicin release	[122]
	Poly(ethylene glycol)-azobenzene-polyethyleneimine-DOPE	siRNA/ Doxorubicin	Cleavage of azobenzene	Cleavage of azobenzene triggers PEG shedding and leads to drug release	[123]
	Methoxy poly(ethylene glycol)-azobenzene-4,4-diamino-poly(d,l-lactide)	Docetaxel	Reductive cleavage of azobenzene	Reductive cleavage leads to structural change of self-assembled micelles and triggers docetaxel release	[124]
	Folic acid-poly(ethylene glycol)-2-nitroimidazole	Sorafenib	Hydrophobic-to-hydrophilic transition of nitro of nitroimidazole	Cohesion of the hydrophobic core of the micelles is weakened; hydrophobic inner core weakens the binding force of the hydrophobic drug, which is more prone to drug leakage and promotes sorafenib release	[125]
	Alendronate-poly(ethylene glycol)-azobenzene-poly-l-lysine	Doxorubicin	Reductive cleavage of azobenzene	Azobenzene cleavage for micelle disassembly triggers doxorubicin release	[126]
	Glucose-poly(ethylene glycol)-azobenzene-IR808-S-S-Paclitaxel	Paclitaxel	Reductive cleavage of azobenzene	Glucose modification promotes cellular uptake; azobenzene cleavage triggers IR808-S-S-PTX release; disulfide bond cleavage triggers paclitaxel release	[127]
Enzyme	Polyethylene glycol-block-poly(acrylic acid)	Doxorubicin	Amidase cleavaging the covalent linked doxorubicin from the micelles	Amidase causes the breakage of amide bond between doxorubicin molecules and polymers, and then triggers disassembly of the micelles to facilitate the doxorubicin release	[128]
	Monomethyl poly(ethylene glycol)-ss-camptothecin/phenylboronic acid-poly(ethylene glycol)-4,4′-(diazene-1,2-diyl)benzoyl-poly(ε-caprolactone)	Camptothecin	Azoreductase	Azoreductase and NADPH facilitates the azobenzene bonds cleavage and GSH facilitate disulfide bond cleavage, which trigger camptothecin release	[129]
	Poly(ethylene glycol)-peptide- polyethyleneimine-1,2-dioleoyl-sn-glycero-3-phosphoethanolamine	Paclitaxel/siRNA	Metalloproteinase 2 cleavage	Polyethyleneimine increases cellular uptake and delivers siRNA and facilitates endosome escape; MMP2 decreases micelles stability and release drugs	[130]
	Methoxypolyethylene glycol amine-glutathione-palmitic acid	Dexamethasone	Glutathione reductase	Glutathione reductase breaks micelles structure and triggers dexamethasone release	[131]
	Poly(ethylene glycol)-b-poly(l-tyrosine)	JQ1	Proteinase K	π–π stacking for efficient and stable encapsulation of JQ1; PTyr degradation by proteinase K triggers JQ1 release	[132]
	D-α-tocopherol polyethylene glycol 3350 succinate-Gly-Pro-Leu-Gly-Val-Arg-doxorubicin /FA-Asp-Glu-Val-Asp-doxorubicin	Doxorubicin	Matrix metalloproteinase (MMP-9); caspase-3	MMP-9 increases micelles endocytosis; caspase-3 increases doxorubicin release	[133]
Thermo	Monomethoxy poly(ethylene glycol)-deoxycholic acid	Estradiol	Lower critical solution temperature (LCST) transition of the micelles facilitating dehydration of the PEG shell	Thermosensitive micelles with a rigid core minimizes the initial burst release of estradiol encapsulated by coating the shell at a temperature above its LCST through the thermal transition	[134]
	Poly(t-butyl acrylate-co-acrylic acid)-b-poly(N-isopropylacrylamide)/chitosan-g-poly(N-isopropylacrylamide)	Doxorubicin	Poly(N-isopropylacrylamide) exerting temperature responsiveness	The pH-sensitive poly(t-butyl acrylate-co-acrylic acid) encapsulates doxorubicin by electrostatic interactions; and the poly(N-isopropylacrylamide) plays the role of aqueous solubilization and responses to temperature changes, and triggers doxorubicin release	[135]
	Poly(N-isopropylacrylamide-b- butylmethacrylat	Adriamycin	Poly(N-isopropylacrylamide) phase transistion	Poly(N-isopropylacrylamide) reverses micelle structure to trigger Adriamycin release	[136]
	P-(N,N-isopropylacrylamide-co-N-hydroxymethylacrylamide)-b-caprolactone	Doxorubicin	Poly(N-isopropylacrylamide) phase transistion	Poly(N-isopropylacrylamide) reverses micelle structure to trigger doxorubicin release	[137]
Magnetic	RGD-poly[(N-isopropylacrylamide-r-acrylamide)-b-L-lactic acid]/oleic acid -SPIONs	Paclitaxel	Magnetic hyperthermia	Hydrophobic PLA segments incorporates SPIONs and paclitaxel, RGD serves as a targeting moiety, and SPIONs concentrate paclitaxel to targeted sites	[138]
	Poly(phenyl isocyanide)s	Doxorubicin/Fe_3_O_4_ nanoparticles	Magnetic hyperthermia	The loading of magnetic Fe_3_O_4_ nanoparticles contributes to the hyperthermia performance; effective drug release due to the morphology change of thermoresponsive poly(phenyl isocyanide)s	[139]

**Table 4:** The clinical trials of polymeric micelles.

Clinical Trial/Drug	Polymeric Carrier	Condition	Status	Phase	Participants	Clinical Trials ID
Pm-Pac/Paclitaxel	PEG-PLA	Non-Small Cell Lung Cancer	Unknown	Phase 3	454	NCT02667743
Genexol-PM/Paclitaxel	PEG-PLA	Taxane-Pretreated Recurrent Breast Cancer	Unknown	Phase 4	90	NCT00912639
	PEG-PLA	Advanced Non-Small Cell Lung Cancer	Completed	Phase 2	276	NCT01023347
	PEG-PLA	Advanced Ovarian Cancer	Unknown	Phase1/2	74	NCT00886717
	PEG-PLA	Advanced Urothelial Cancer Previously Treated with Gemcitabine and Platinum	Completed	Phase 2	37	NCT01426126
	PEG-PLA	Advanced Pancreatic Cancer	Completed	Phase 2	43	NCT00111904
	PEG-PLA	Advanced Hepatocelluar Carcinoma After Failure of Sorafenib	Terminated	Phase 2	5	NCT03008512
	PEG-PLA	Advanced Non-small-cell Lung Cancer	Completed	Phase 2	45	NCT01770795
	PEG-PLA	Gynecologic Cancer (Adult Solid Tumor)	Unknown	Phase 1	18	NCT02739529
	PEG-PLA	Pancreatic Cancer	Completed	Phase 1	18	NCT00882973
	PEG-PLA	Metastatic or Locally Recurrent Breast Cancer	Completed	N/A	111	NCT02064829
NANOXEL-M/Docetaxel	PEG-PLA	Esophageal Squamous Cell Carcinoma	Unknown	Phase 2	38	NCT03585673
	PEG-PLA	Recurrent or Metastatic Head and Neck Squamous Cell Carcinoma	Unknown	Phase 2	31	NCT02639858
NC-6004/Cisplatin	PEG-Poly (glutamic acid)	Recurrent and/or Metastatic Squamous Cell Carcinoma of the Head and Neck	Terminated	Phase 1	4	NCT02817113
	PEG-Poly (glutamic acid)	Locally Advanced or Metastatic Pancreatic Cancer	Completed	Phase 3	310	NCT02043288
NK105/Paclitaxel	PEG-Polyaspartate	Metastatic or Recurrent Breast Cancer	Completed	Phase 3	436	NCT01644890
NC-4016/Oxaliplatin	PEG-Poly (glutamic acid)	Advanced Solid Tumors or Lymphoma	Completed	Phase 1	34	NCT03168035
NC 6300/Epirubicin	PEG-Polyaspartate	Advanced Solid Tumors or Advanced, Metastatic, or Unresectable Soft Tissue Sarcoma	Unknown	Phase1b/2	150	NCT03168061
NK012/ SN-38	PEG-Poly (glutamic acid)	Advanced Solid Tumors Followed by a Dose Expansion Phase in Patients With Metastatic Colorectal Cancer	Completed	Phase 1	35	NCT01238939
	PEG-Poly (glutamic acid)	Sensitive Relapsed and Refractory Relapsed Small-Cell Lung Cancer	Completed	Phase 2	72	NCT00951613
	PEG-Poly (glutamic acid)	Locally Advanced Non-Resectable and Metastatic Breast Cancer Patients With Triple Negative Phenotype	Completed	Phase 2	61	NCT00951054
	PEG-Poly (glutamic acid)	Refractory Solid Tumors	Completed	Phase 1	39	NCT00542958
	PEG-Poly (glutamic acid)	Advanced Solid Tumors Followed by a Dose Expansion Phase in Patients With Triple Negative Metastatic Breast Cancer	Completed	Phase 1	4	NCT01238952
BIND-014/Docetaxel	PEG-PLA	Metastatic Castration-Resistant Prostate Cancer	Completed	Phase 2	42	NCT01812746
	PEG-PLA	Non-Small Cell Lung Cancer	Completed	Phase 2	64	NCT01792479
	PEG-PLA	Advanced or Metastatic Cancer	Completed	Phase 1	58	NCT01300533
	PEG-PLA	KRAS Mutation Positive or Squamous Cell Non-Small Cell Lung Cancer	Completed	Phase 2	69	NCT02283320
	PEG-PLA	Urothelial Carcinoma, Cholangiocarcinoma, Cervical Cancer and Squamous Cell Carcinoma of the Head and Neck	Terminated	Phase 2	73	NCT02479178
Paclitaxel Micelles	Micelles (polymer unknown)	Advanced Solid Tumor	Not yet recruiting	Phase 1	98	NCT04778839
Docetaxel Polymeric Micelles	Micelles (polymer unknown)	Advanced Malignant Solid Tumors	Not yet recruiting	Phase 2	110	NCT05254665
Cisplatin Micelles (HA132)	Micelles (polymer unknown)	Advanced Malignant Solid Tumors	Not yet recruiting	Phase 1/2	126	NCT05478785
PLZ4-coated paclitaxel micelles (PPM)	PEG-Cholic acid	Non-myoinvasive Bladder Cancer	Recruiting	Phase 1	29	NCT05519241

Abbreviation: PEG-PLA: poly(ethylene glycol)-poly(lactide).

## Data Availability

Data sharing not applicable.
